# The staphylococcal type VII secretion system protein EsxC impacts daptomycin sensitivity through controlling bacterial cell envelope integrity

**DOI:** 10.1128/jb.00380-25

**Published:** 2026-01-12

**Authors:** Victoria Smith, Robeena Farzand, Giridhar Chandrasekharan, Kate E. Watkins, Ramon Garcia Marset, Jeannifer Yap, Sebastien Perrier, Arnaud Kengmo Tchoupa, Meera Unnikrishnan

**Affiliations:** 1 Division of Biomedical Sciences, Warwick Medical School, University of Warwick 2707, Coventry, United Kingdom; 2 Department of Chemistry, University of Warwick 2707, Coventry, United Kingdom; 3 Department of Chemistry and Warwick Medical School, University of Warwick 2707, Coventry, United Kingdom; University of Notre Dame, Notre Dame, Indiana, USA

**Keywords:** type VII secretion system, membrane integrity, membrane fluidity, daptomycin

## Abstract

The human pathogen *Staphylococcus aureus* encodes a specialized type VII secretion system (T7SS), which plays an important role in bacterial virulence during infection. However, the functions of the T7SS during infection and in bacterial physiology remain unclear. Here, we demonstrate that *S. aureus* strains lacking a T7SS effector, EsxC (Δ*esxC*), were highly sensitive to the important last resort drug, daptomycin, as well as other membrane-targeting antibiotics, including gramicidin and bithionol. To understand how EsxC mediates increased antibiotic sensitivity, we investigated the Δ*esxC* cell envelope. Scanning electron microscopy analysis of the *esxC* mutant revealed a distinct cell surface morphology. Δ*esxC* also displayed a decrease in membrane fluidity, altered membrane protein profiles, and altered cell wall synthesis. The *esxC* mutant membranes demonstrated an increased negative charge compared to the WT, which was associated with an enhanced daptomycin binding. We then showed that calcium ions, which are required for daptomycin activity, impacted growth of Δ*esxC* and sensitivity to daptomycin, suggesting that EsxC is involved in modulating calcium binding to the membrane. Furthermore, the *esxC* mutant displayed a heightened susceptibility to daptomycin during intracellular infection and in a murine skin infection model. Thus, our data show that the T7SS protein EsxC, in association with other T7SS proteins, contributes to the sensitivity of *S. aureus* to membrane-acting drugs, such as daptomycin, through modulation of cell membrane integrity.

**IMPORTANCE:**

Type VII secretion system (T7SS) has a range of functions in bacteria, including specific roles in bacterial physiology, including DNA uptake, membrane integrity, and bacterial development. In *S. aureus,* T7SS has been shown to be critical for bacterial virulence, intra-species competition, and in host cell interactions, although their functions in bacterial physiology are not clear. Here, we report a role of the staphylococcal T7SS component EsxC in the modulation of the cell membrane and surface integrity, likely in association with co-dependent effectors, which impacts the activity of membrane targeting drugs like daptomycin. Our data indicate that targeting the T7SS could provide a new approach to enhancing activity of existing therapeutic agents.

## INTRODUCTION

*Staphylococcus aureus* is a highly versatile pathogen and a commensal bacterium that causes infections in both humans and animals. Staphylococcal infections can be nosocomial or community-acquired and can range from mild skin abscesses to severe life-threatening infections, including septic shock and bacteremia (1). *S. aureus* infections place a huge cost burden on healthcare sectors across the world, especially due to the occurrence of antibiotic-resistant strains, such as methicillin-resistant *S. aureus* (MRSA) and strains with varying levels of vancomycin resistance (2, 3).

*S. aureus* possesses an array of surface and secreted factors that help the bacterium colonize the host and establish an infection (4). One such virulence-associated factor is the type VII secretion system (T7SS), which is widely found in Gram-positive bacteria (5). The T7SSb in *S. aureus* and other firmicutes is distinct from the systems in mycobacteria (T7SSa), although both are characterized by small substrates containing the WXG motif, such as EsxA and EsxB (6, 7). The T7SS in *S. aureus* is modular in nature, with heterogeneous expression between strains (8, 9). Typically, in commonly studied *S. aureus* strains, including USA300, Newman, and RN6390, the T7SS locus comprises integral membrane proteins (EsaA, EssA, EssB, and EssC), cytosolic proteins (EsaB and EsaG), several secreted substrates (EsxA, EsxB, EsxC, EsxD, and EsaD), and chaperones (EsaE) (7, 10). EssC is the central transporter, which is responsible for export of substrates and co-dependent export of secreted substrates, has been reported (6, 11). While specific interactions between the different T7SS components have been described, the overall structure of the secretion apparatus is poorly defined for the *S. aureus* T7SS (10, 11). Assembly of a functional system is dependent on the membrane microdomain protein, flotillin A (FloA) (12).

T7SS has been reported to play a role in the modulation of intraspecies competition. A T7SS substrate, EsaD, with nuclease activity mediates killing of *S. aureus* strains that lack the anti-toxin EsaG (13). An LXG-domain containing protein, TspA, encoded distally to the T7SS cluster shows T7SS-dependent toxic activity, which can be neutralized by an antitoxin TsaI (14). Recent work has shown that the secretion of the nuclease toxin EsaD was facilitated by EsxD, EsxC, and EsxB, which formed a pre-secretion complex (15). Some T7 components modulate host interactions during infection; EsxA modulates host cell death, and EsaE elicits cytokine production during infection (16, 17). However, effectors of this system are yet to be defined in many *S. aureus* strains, and their specific functions remain elusive.

T7SS in other bacterial species has been described to support bacterial functions like DNA transfer, membrane integrity, and spore development, although for *S. aureus*, it is not clear if T7SS is important in staphylococcal physiology (18–20). The T7SS substrate EsxC is a small WXG-like protein, expressed and important during persistent murine *S. aureus* infection (21). Our recent study showed that a mutant lacking EsxC was more sensitive to unsaturated host-derived fatty acids (22). T7SS defects compromised membrane integrity and induced oxidative stress in the presence of antimicrobial fatty acids, indicating a role for these proteins in membrane homeostasis. Here, we report that the absence of EsxC causes an increased sensitivity to membrane targeting drugs like daptomycin, both during infection *in vitro* and *in vivo*. We demonstrate distinct effects on cell surface morphology, membrane fluidity, and charge, indicating a role for T7SS in controlling membrane integrity potentially through modulating calcium binding. Our data suggest that targeting the T7SS could augment activities of membrane-acting drugs like daptomycin.

## RESULTS

### A mutant lacking T7SS effector EsxC is more sensitive to membrane-acting drugs

**Fig 1:**
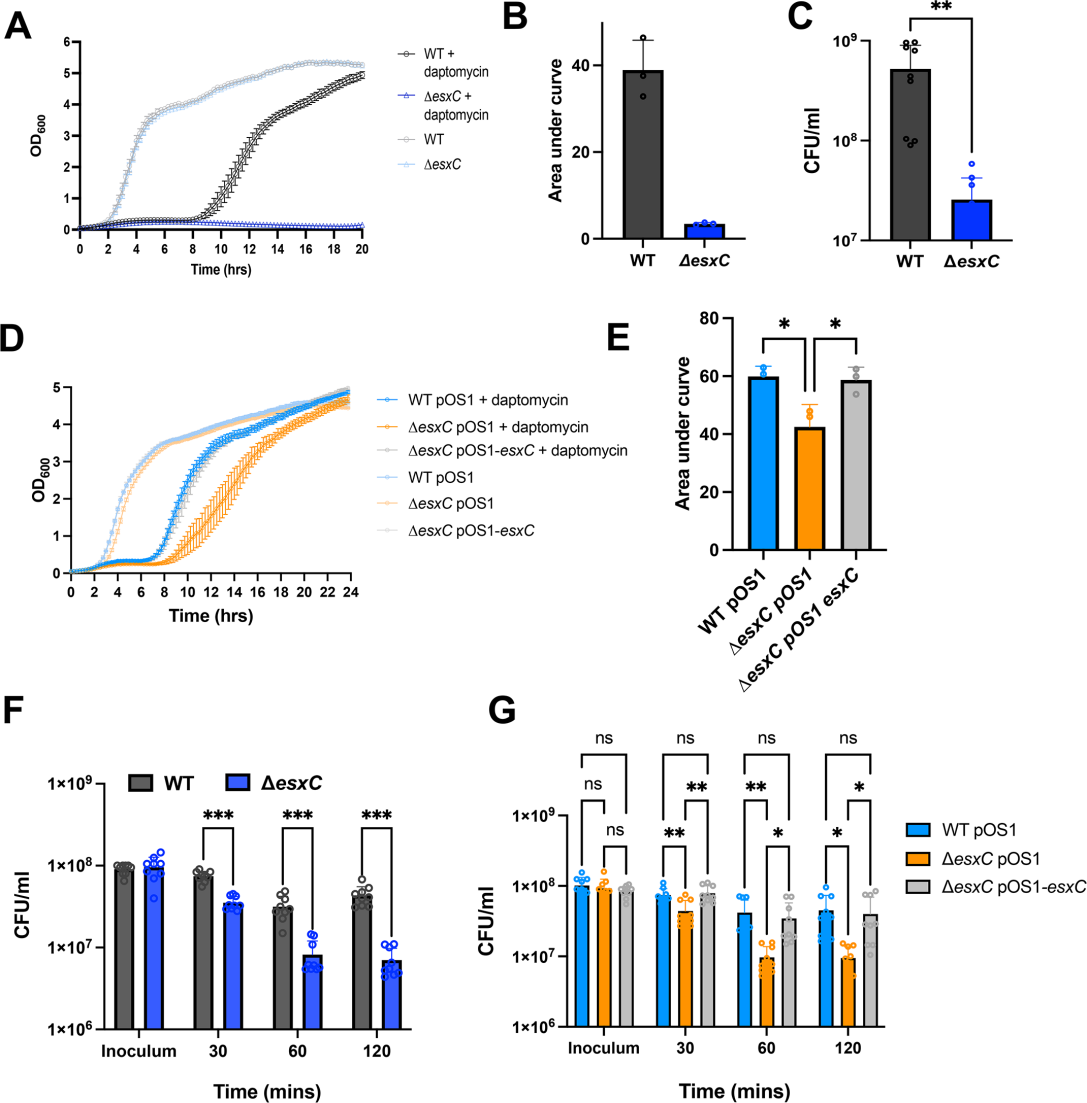
An *esxC* mutant is highly sensitive to daptomycin. (**A**) Growth curves of *S. aureus* WT USA300 JE2 and Δ*esxC* in TSB in the absence or presence of 5 μg/mL daptomycin and 1 mM CaCl_2_. Mean ± standard error of the mean (SEM) is shown, *N* = 3 (biological replicates). AUC was calculated for the strains grown in the presence of daptomycin (**B**). (**C**) CFU/mL was enumerated after 8 h daptomycin treatment. *N* = 3 (biological replicates), mean ± standard deviation (SD), ***P ≤ 0.001 using an unpaired *t*-test. (**D**) Growth curves of USA300 JE2 WTpOS1, Δ*esxC*pOS1, and complemented strain, Δ*esxC*pOS1-*esxC* in TSB supplemented with 5 μg/mL daptomycin and 1 mM CaCl_2_. Mean ± SEM shown, *N* = 3 (biological replicates); AUC was calculated for the strains grown in the presence of daptomycin (**E**), mean ± SD **P ≤ 0.05 using a one-way ANOVA with Tukey’s multiple comparison test. (**F**) CFU/mL of WT USA300 JE2 and Δ*esxC* was determined from a killing assay in the presence of 10 μg/mL daptomycin and 1 mM CaCl_2_ at different time points. *N* = 3 (biological replicates), (**G**) CFU/mL from killing assay using WT USA300 JE2, Δ*esxC,* and Δ*esxC* complemented strains in the absence or presence of 10 μg/mL daptomycin and 1 mM CaCl_2_ at different timepoints, mean ± SD, *N* = 3, *P ≤ 0.05, **P ≤ 0.01, ***P ≤ 0.001 using a two-way ANOVA with a Tukey’s multiple comparison test for (**F**) and (**G**).

**Fig 2:**
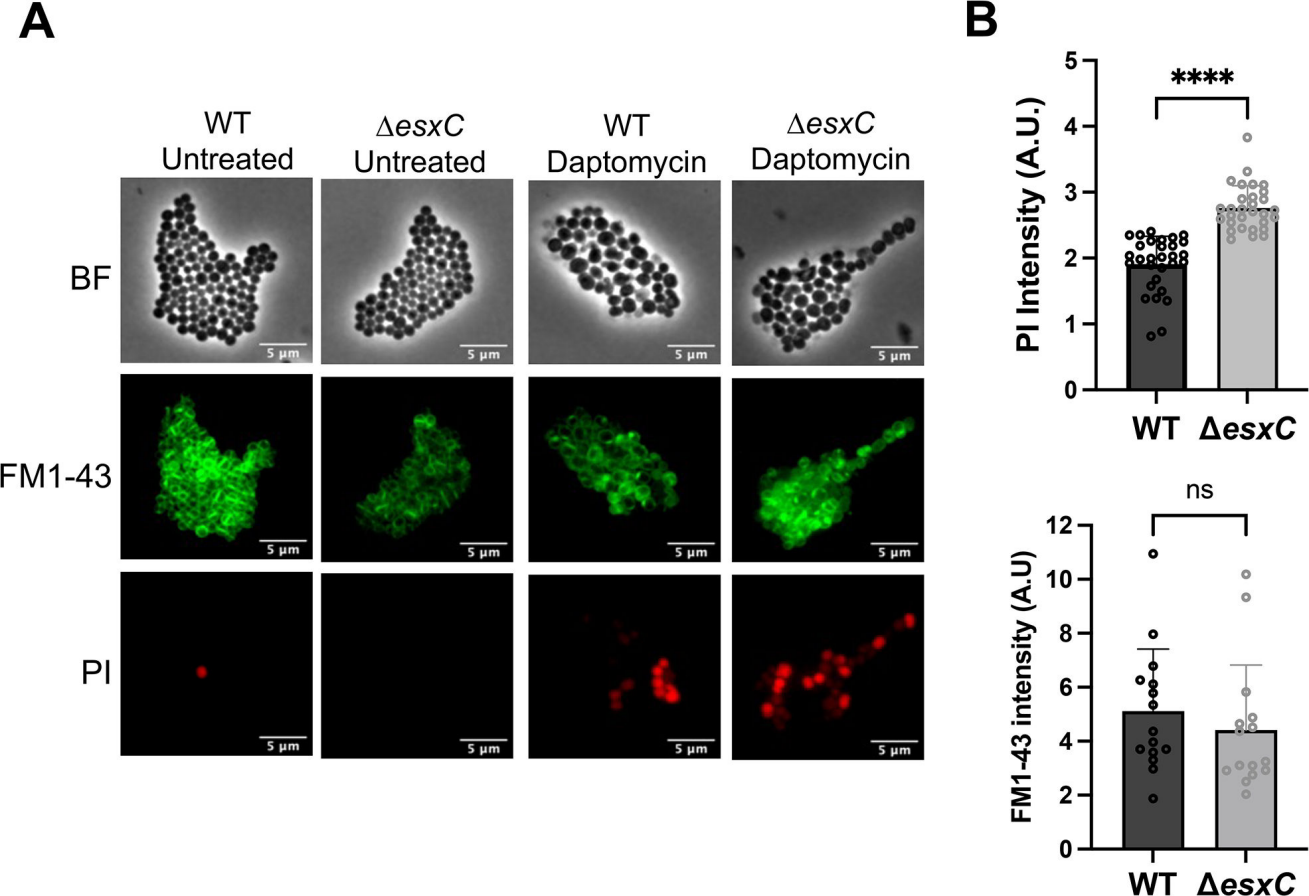
Membrane permeability of the *esxC* mutant in the presence of daptomycin. (**A**) Representative fluorescent micrographs of *S. aureus* USA300 JE2 WT and Δ*esxC* treated with daptomycin. Cell membranes were stained by FM1-43 and imaged using FITC filter channel, PI was imaged using Texas red filter channel. (**B**) Images of daptomycin-treated samples in (**A**) were quantified using FIJI. Mean fluorescence intensity was quantified from 5–10 images from each experiment and normalized to the number of cells in the field of view. Data presented are from three independent experiments. ****P < 0.0001 using an unpaired t test with Welch’s correction. ns, non significant.

The cyclic lipopeptide antibiotic, daptomycin, is a key drug used to treat resilient *S. aureus* infections. Daptomycin binds to the cell membrane in a calcium-dependent manner (23). As our previous studies indicated a potential impact of T7SS, in particular the effector EsxC, on membrane homeostasis (22), we investigated whether the *S. aureus* USA300 JE2 mutants lacking the T7SS substrate EsxC (Δ*esxC*) respond differently to membrane-acting drugs like daptomycin. When cultured in the presence of 5 μg/mL daptomycin, growth of the wild-type (WT) USA300 JE2 strain was delayed, but growth of USA300 JE2 Δ*esxC* was significantly impaired compared to the WT (Fig. 1A and B). No differences in growth rates were seen between the strains in tryptic soy broth (TSB) without antibiotic. Further, a 10-fold decrease in colony-forming unit counts for the mutant was also observed in the presence of daptomycin (Fig. 1C). Complementing Δ*esxC* with pOS1-*esxC* restored WT phenotype (Fig. 1D and E), although daptomycin did not inhibit the pOS1 empty plasmid-bearing Δ*esxC* to the same extent as Δ*esxC* without plasmid. This could be attributed to differences in growth profiles of pOS1 containing WT and mutant strains, possibly due to effects of chloramphenicol exposure prior to performing growth assays. Next, in a daptomycin killing assay where logarithmic phase bacteria were treated for 2 h with daptomycin, Δ*esxC* was more sensitive to daptomycin compared to the WT, with the Δ*esxC* pOS1-*esxC* strain reversing the daptomycin sensitivity (Fig. 1F and G). When we stained bacteria with propidium iodide (PI), a fluorescent intercalating agent that cannot pass through the membranes of viable cells, cytosols of daptomycin-treated Δ*esxC* showed a higher intensity of PI compared to the WT, as quantified by fluorescence microscopy (Fig. 2A and B). Staining with the lipid staining dye FM1-43 was of similar intensity for WT and Δ*esxC* (Fig. 2A and B). Thus, Δ*esxC* demonstrated increased membrane permeability in the presence of daptomycin.

To investigate whether other T7SS effectors had similar effects, we studied strains lacking substrates EsxA and EsxB using mutants in USA300 LAC and Newman backgrounds. Growth defects were observed in USA300 LAC and Newman Δ*esxA* mutants compared with the respective WT strains, while Δ*esxB* mutants did not appear to have a growth defect (Fig. S1A through D). The Newman strain was more sensitive than USA300 JE2 to daptomycin, with growth delayed until ~14 h. Furthermore, a USA300 JE2 mutant lacking another T7SS substrate, EsxD, did not display any growth defects in the presence of daptomycin (Fig. S1E and F). Also, a USA300 JE2 strain lacking the central transporter EssC (Δ*essC*) responsible for transport of T7SS effectors, as expected, showed significantly impaired growth compared with the WT (Fig. S1G and H). Complementation of daptomycin sensitivity was seen for Δ*esxA* and Δ*essC* (Fig. S1K through N). Furthermore, Δ*esxC* and Δ*essC* mutants in a *S. aureus* RN6390 background were more susceptible than the WT to daptomycin, although bacterial growth initiated much later (~14 h) compared with the JE2 strain (~8 h) (Fig. S1I and J).

**Fig 3:**
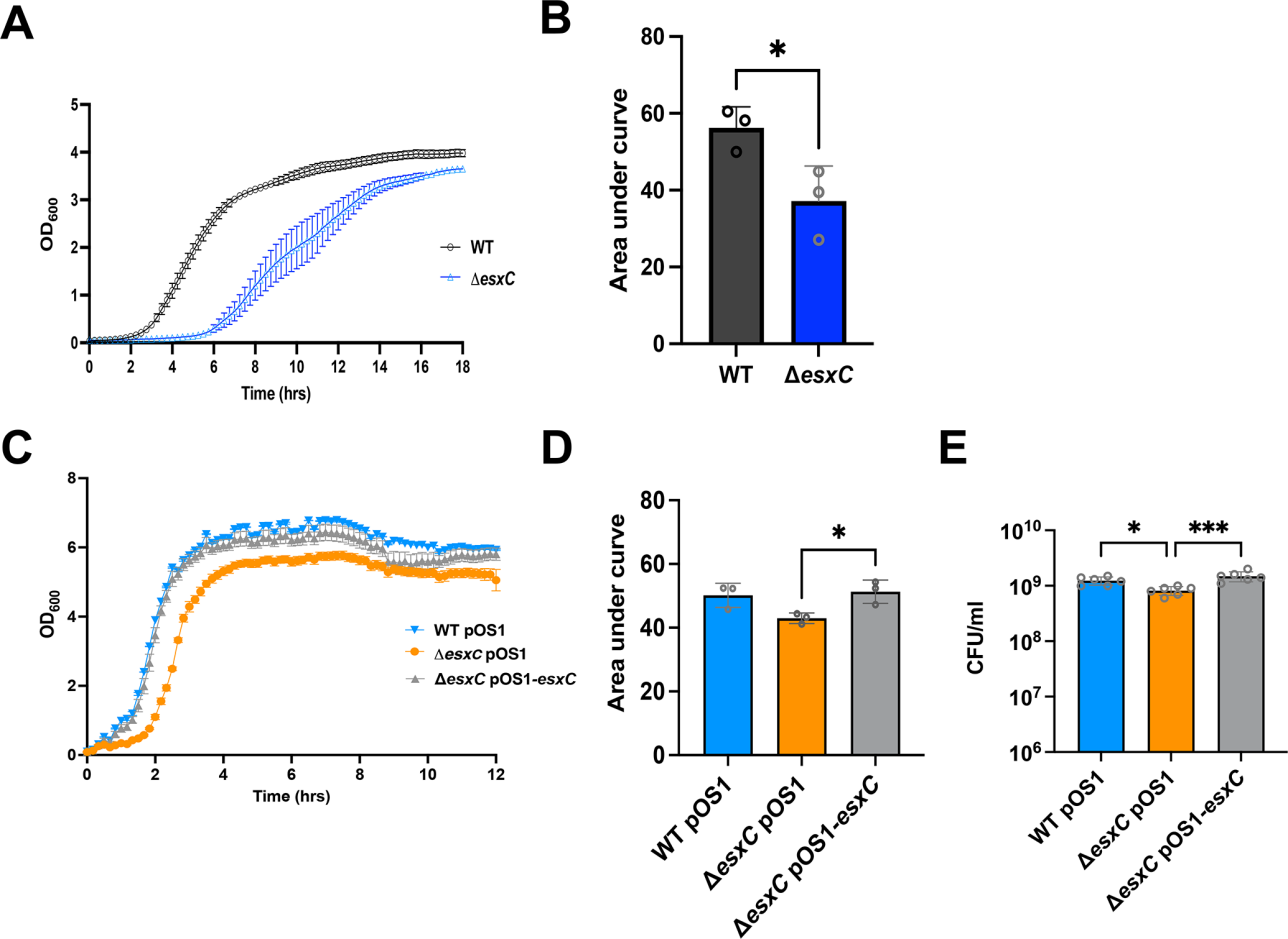
The *esxC* mutant shows higher sensitivity to other membrane-acting drugs. Growth curves of USA300 JE2 WT and Δ*esxC* (**A**) and (**C**) WTpOS1, Δ*esxC* pOS1, and Δ*esxC*pOS1*esxC* plasmid-complemented strains in the presence of 2 μg/mL gramicidin, mean ± SEM, *N* = 3 (biological replicates). (**B** and **D**) Area under curves (AUC) were calculated, and the mean AUC ± SD was plotted for growth curves in (**A**) and (**C**), **P = 0.001 using an ordinary one-way ANOVA. (**E**) CFU/mL was enumerated after 4 h gramicidin treatment. *N* = 3 (biological replicates), mean ± SD is shown, ***P = 0.004 using a one-way ANOVA and Sidak’s multiple comparisons test.

To determine if Δ*esxC* was sensitive to other membrane-targeting antimicrobials, gramicidin, a polypeptide antimicrobial peptide that forms ion channels in the cell membrane (24), and bithionol, an inhibitor of soluble adenylyl cyclase and its membrane-disrupting activity, were tested (25). The WT JE2 strain did not show any growth defects when treated with 2 μg/mL gramicidin; however, the Δ*esxC* mutant displayed slower growth (Fig. 3A and B). Complementation reversed the phenotype, but as with daptomycin, the plasmid containing the Δ*esxC* mutant strain showed a reduced sensitivity as compared with the Δ*esxC* mutant (Fig. 3C through E). A slightly slower growth was also seen for the mutant in the presence of 1 μg/mL bithionol compared with the WT (Fig. S2A through C). Finally, this effect appears to be specific to membrane-acting drugs as drugs targeting the cell wall (vancomycin, oxacillin) or DNA replication (ciprofloxacin, mitomycin) did not differentially affect the growth of the Δ*esxC* (or Δ*essC*) as compared with the WT (Fig. S2D through G).

### Δ*esxC* displays decreased membrane fluidity and altered membrane protein profiles

**Fig 4:**
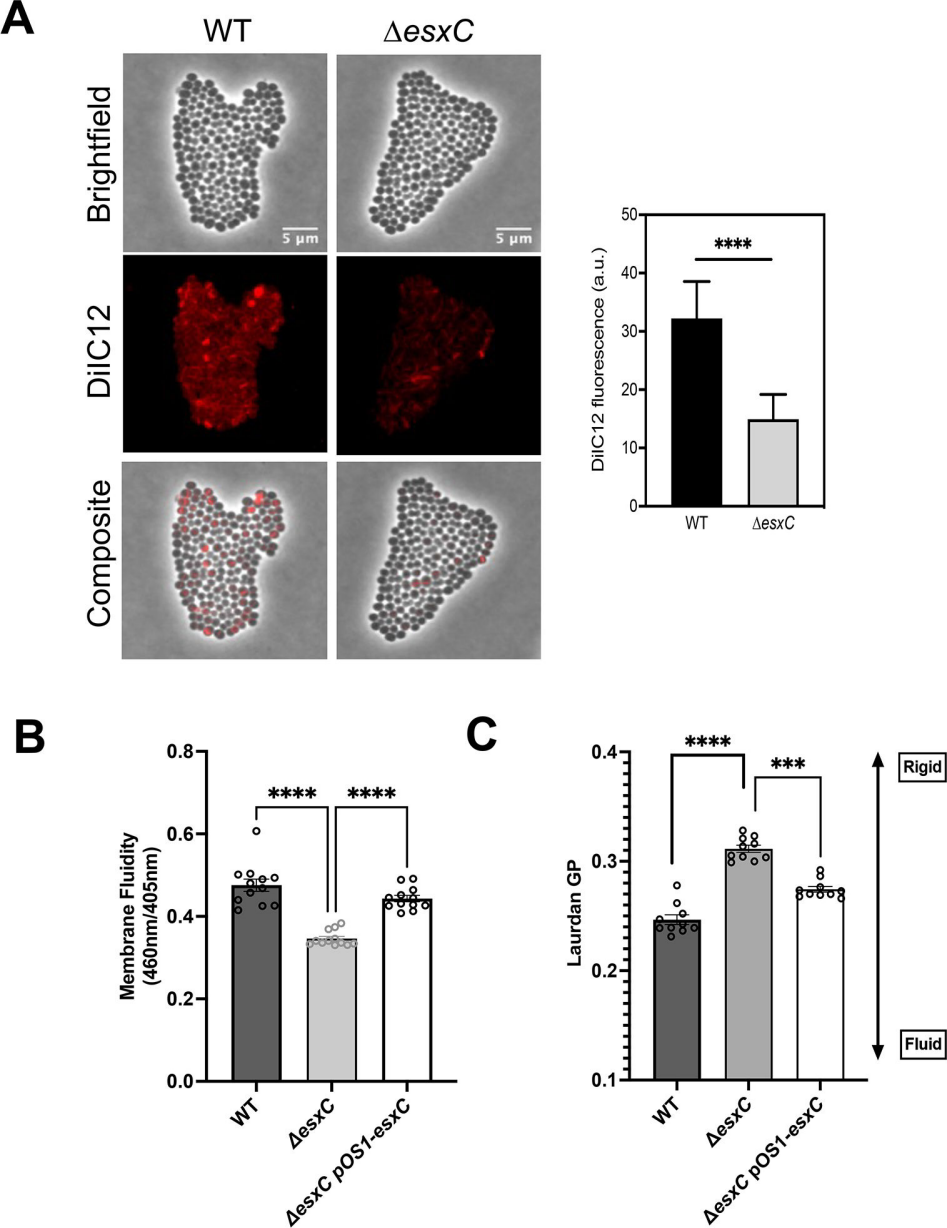
EsxC affects membrane fluidity. (**A**) Widefield micrographs of *S. aureus* WT USA300 JE2 and Δ*esxC* after growth in TSB to OD_600_ of 1.0 in the presence of the lipophilic dye DiIC12. Images are representative of 4 independent experiments. The DiIC12 fluorescence of 80 bacterial clusters from different fields per strain was quantitated with ImageJ. Means ± SD are shown, *N* = 4 (biological replicates); ****P < 0.0001, using an unpaired *t*-test. (**B**) The membrane fluidity of *S. aureus* WT USA300 JE2, Δ*esxC,* WTpOS1, Δ*esxC* pOS1, and Δ*esxC*pOS1*esxC* as measured with a pyrene decanoic acid staining-based assay, mean ± SEM is shown, *N* = 6 (biological replicates), * indicates *P* < 0.05 using a one-way ANOVA with Tukey’s multiple comparisons test. (**C**) USA300 JE2 WT, Δ*esxC,* WTpOS1, Δ*esxC* pOS1, and Δ*esxC*pOS1*esxC* were grown to logarithmic phase in TSB at 37°C prior to staining with Laurdan. To assess membrane fluidity, Laurdan generalized polarization (GP) was calculated using the equation (I_460_ – I_500_)/ (I_460_ I_500_), where I refers to the fluorescence intensity at the indicated emission wavelength, mean ± SD is shown, *N* = 3 (biological replicates), ***P <0.0001 using a one-way ANOVA with Tukey’s multiple comparisons test.

Changes in membrane fluidity can affect membrane function and antibiotic sensitivity. We employed multiple assays to assess the membrane fluidity of the Δ*esxC* mutant. The WT strain stained with a fluorescent membrane dye DiI12C, which detects regions of increased fluidity in bacteria (26), showed a few fluid regions with uneven membrane staining, as reported previously. In contrast, the Δ*esxC* mutant displayed weak membrane staining (Fig. 4A), which may indicate a decrease in membrane fluidity. In an alternate membrane fluidity assay using pyrene decanoic acid, an excimer-forming lipid (27), compared with the WT, the Δ*esxC* mutant membranes showed a mild but statistically significant increase in rigidity, which was reversed upon complementation of *esxC* (Fig. 4B). The plasmid containing WT and mutant strains did not behave differently, unlike growth curve experiments in Fig. 1 and 3. Finally, membrane fluidity measurements using the fluorescent dye Laurdan (6-dodecanoyl-2-dimethylaminonaphthalene), which is incorporated into the membrane bilayer (28), revealed a lower average generalized polarization value of 0.33 for WT compared with 0.42 for Δ*esxC*, which was reversed in Δ*esxC* pOS1*esxC*, suggesting that the membrane of the Δ*esxC* mutant is more rigid in comparison to that of the WT (Fig. 4C). Overall, the data indicate that EsxC contributes to modulation of *S. aureus* membrane fluidity.

Previously, we reported that Δ*esxC* and Δ*essC* had distinct total protein profiles with and without treatment with linoleic acid (22). Given the changes seen in bacterial membranes in the absence of EsxC, we examined global changes in the protein content in the membrane. Membrane fractions extracted from logarithmic phase (OD_600_ = 3.0) cultures of WT and Δ*esxC* were analyzed by nanoLC-ESI-MS/MS. Thirteen proteins were altered in abundance [log2 (fold change) = 1.0, *P* < 0.05] in Δ*esxC* in relation to the WT (Table 1). Membrane proteins were increased in abundance, and EsaD, a T7SS nuclease, was decreased significantly in the membranes of Δ*esxC*. A secretome analysis from culture supernatants showed a lower abundance of substrates EsxA, EsaD, and LXG toxin TspA (A0A0H2XH53) (14) in the Δ*esxC* compared with WT (Table S1). Thus, the *esxC* mutant shows distinct changes to the overall membrane protein profiles and secretion of certain T7SS substrates appears to be co-dependent on substrate EsxC (15).

**TABLE 1:** Proteins significantly changed in Δ*esxC* mutant membrane preparations relative to WT USA300 JE2

Uniprot ID	log_2_ fold change	Adjusted *P*-value	Description
A0A0H2XJR2	−5.46	6.21E-08	Pyridine nucleotide-disulfide oxidoreductase
A0A0H2XJ15	−5.34	3.80E-09	TPR domain protein
A0A0H2XIV9	−5.33	1.49E-07	T7SS protein, EsaD
A0A0H2XJ90	−4.82	6.92E-09	D-isomer specific 2-hydroxyacid dehydrogenase family protein
A0A0H2XF36	3.62	2.14E-08	Uncharacterized protein
A0A0H2XJV3	3.67	2.26E-08	ABC transporter, ATP-binding protein
A0A0H2XGS2	3.70	1.71E-08	Putative sensor histidine kinase
A0A0H2XG87	4.01	1.59E-08	Bifunctional ligase/repressor BirA
A0A0H2XH85	4.14	1.04E-08	Cobalamin synthesis protein/P47K family protein
A0A0H2XER9	4.38	9.36E-09	LysR family regulatory protein
A0A0H2XEB7	5.25	2.39E-07	Integral membrane protein
A0A0H2XJ96	5.27	1.04E-08	NADH-dependent flavin oxidoreductase
A0A0H2XHU5	5.97	2.55E-09	Putative membrane protein

### EsxC contributes to altered cell surface morphology and decreased rate of cell wall synthesis

**Fig 5:**
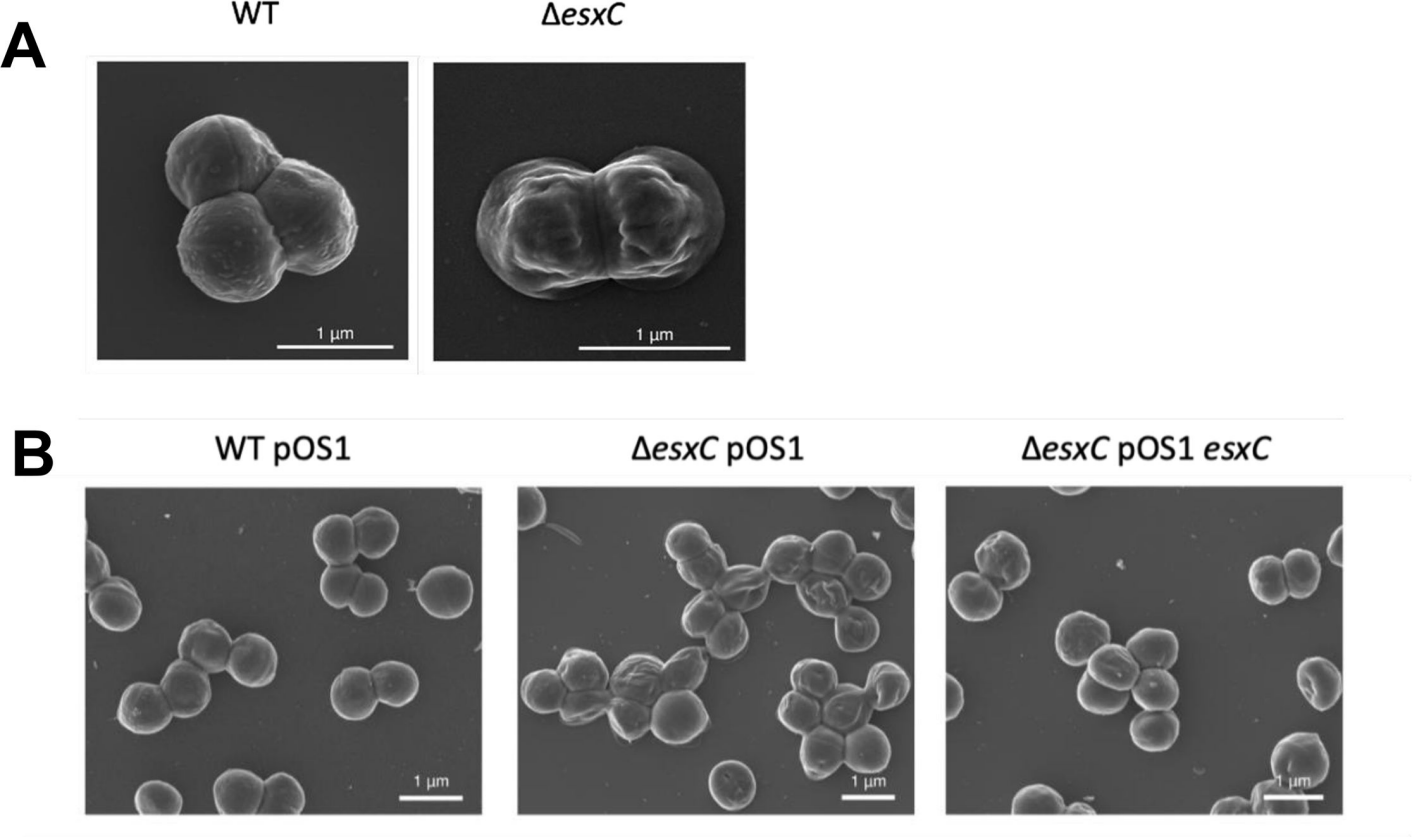
The *esxC* mutant displays a distinct surface morphology. (**A**) Representative SEM images of *S. aureus* WT USA300 JE2 and Δ*esxC* grown to early logarithmic phase. (**B**) Representative SEM images of *S. aureus* USA300 JE2 WT pOS1, Δ*esxC* pOS1, and Δ*esxC* pOS1-*esxC* grown to early logarithmic phase.

As EsxC appeared to be involved in membrane function, we next examined the cell surface of Δ*esxC* using scanning electron microscopy. The cell surface of early logarithmic phase WT showed a typical smooth spherical shape; however, Δ*esxC* appeared deflated with dents or hollows (Fig. 5A). The WT phenotype was restored in the Δ*esxC* pOS1-*esxC* complemented strain (Fig. 5B). Interestingly, this cell surface defect was not seen in Δ*esxD* (Fig. S3). *S. aureus* cell surface alterations were seen in USA300 (LAC) and Newman strains lacking EsxA (Δ*esxA)* but not in Δ*esxB* (Fig. S3). However, RN6390 strains lacking *esxC* showed very little surface defects (Fig. S3). Finally, the JE2 transporter mutant Δ*essC* showed defects similar to Δ*esxC* (Fig. S3). Differences seen in surface morphologies were not due to growth rate differences, as growth rates of all the mutant strains in TSB were similar to that of their respective WT counterparts (Fig. S1). Our data suggest that the cell envelopes of mutants lacking specific T7SS components were altered, potentially due to a defect in the cell membrane and/or cell wall structure.

**Fig 6:**
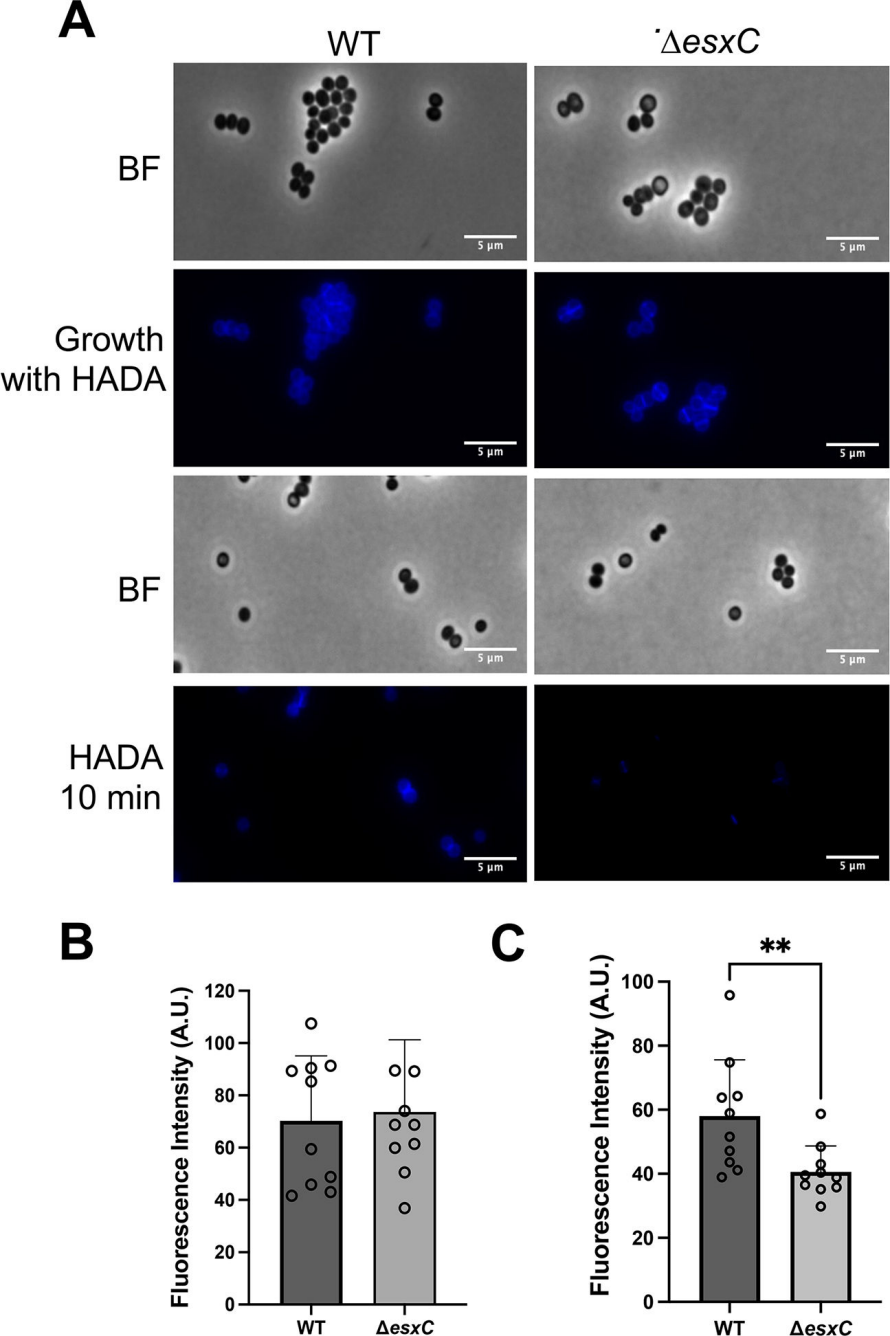
EsxC impacts cell wall synthesis. (**A**) Representative widefield micrographs of *S. aureus* WT USA300 JE2 and Δ*esxC* either grown to logarithmic phase in the presence of 25 μM HADA or after 10-min treatment with HADA once OD_600_ of 1 was reached. (**B**) Quantification of fluorescence in cells grown to logarithmic phase with HADA. (**C**) Quantification of fluorescence in cells treated with HADA for 10 min. Quantification was performed on five images each from three independent experiments. **P< 0.01 and using an unpaired *t*-test.

Transmission electron microscopy was used to investigate if the surface structure changes were associated with defective cell wall structure or synthesis, but no clear differences in cell wall thickness were seen between strains (Fig. S4). To study whether peptidoglycan synthesis and accumulation were affected in the T7SS mutants, the fluorescent D-amino acid, 7-hydroxycoumarincarbonylamino-D-alanine (HADA), was used (29). No differences were observed in the cells, which were previously grown to log phase and supplemented with HADA, a fluorescent D-amino acid (Fig. 6A and B). However, the addition of HADA during exponential phase growth revealed a faster accumulation of HADA in the WT compared with Δ*esxC* (Fig. 6A and C). This suggests that Δ*esxC* displayed a decreased rate of new peptidoglycan synthesis.

### Δ*esxC* displays an increased negative cell surface charge and increased binding of daptomycin

**Fig 7:**
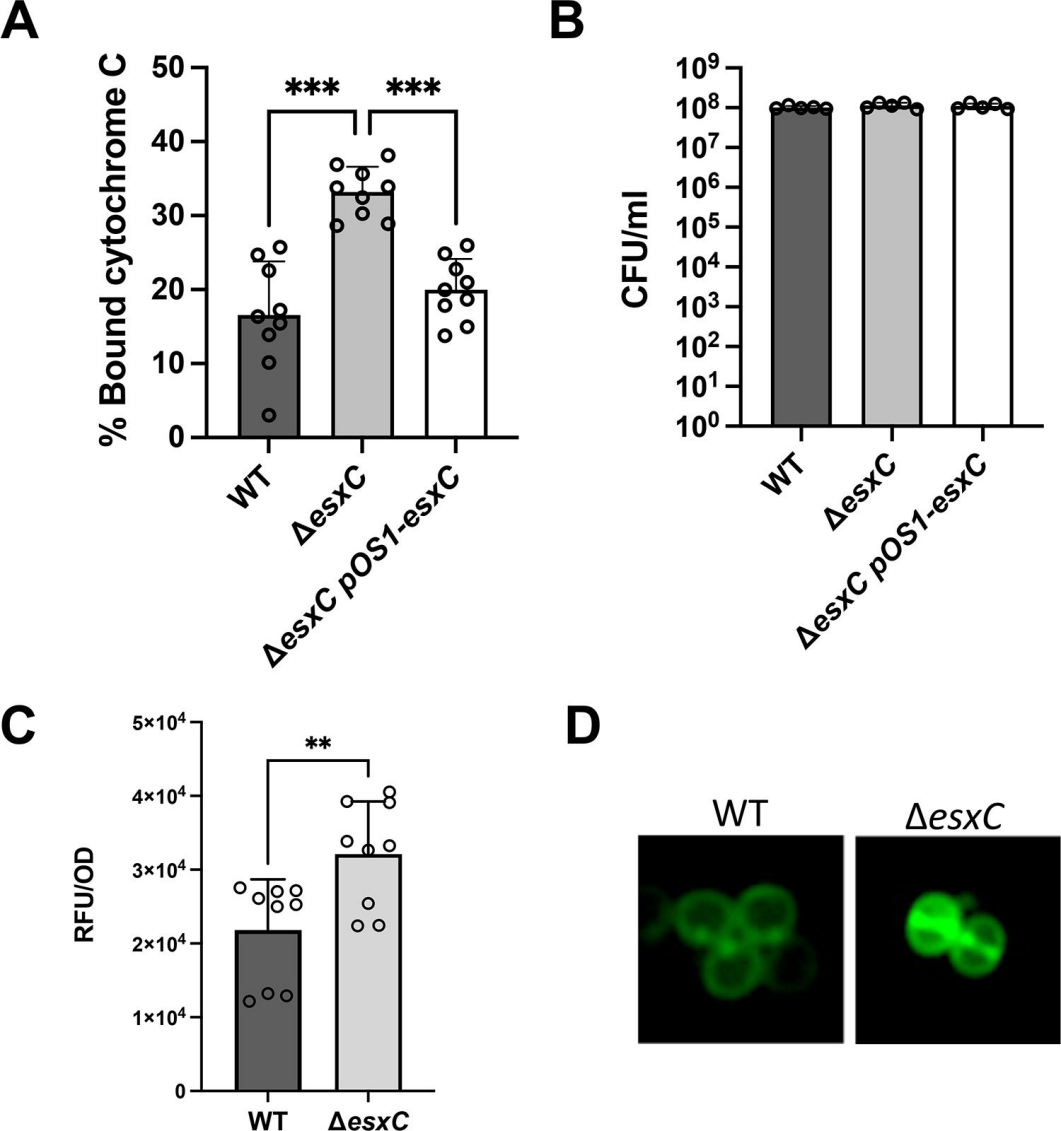
*EsxC* mutant membranes are more negatively charged. (**A**) Quantitative binding assay of cytochrome C to USA300 JE2 WT, Δ*esxC,* and Δ*esxC*pOS1-*esxC.* The CFU of the starting inoculum was calculated for each mutant (**B**). Mean ± SD shown, *N* = 3 (biological replicates), ***P < 0.001 using an ordinary one-way ANOVA with a Tukey’s multiple comparisons test. (**C**) Fluorescence measurements of daptomycin-BODIPY binding to *S. aureus* WT USA300 JE2 and Δ*esxC.* The excitation wavelength was 488 nm, and emission wavelength was 530 nm. *N* = 3 (biological replicates), mean ± SD. **P = 0.0067, using an unpaired *t*-test. (**D**) Representative micrographs of daptomycin-BODIPY binding to *S. aureus* WT and Δ*esxC* are shown*.*

As we observed cell surface defects and changes to peptidoglycan synthesis, we tested if the deletion of *esxC* could alter other cell surface properties like surface charge. A cytochrome binding assay was employed to measure surface charge; the binding of cytochrome C, a highly positively charged protein, is directly proportional to the net negative surface charge of *S. aureus* cells (30). A significantly higher level of binding of cytochrome C to the cell surface of Δ*esxC* and Δ*esxA* was observed compared with the respective WT (Fig. 7A; Fig. S5), suggesting that the cell surfaces of Δ*esxC* and Δ*esxA* were more negatively charged. Much lower levels of cytochrome C bound to USA300 LAC WT and Δ*esxB,* suggesting these cell surfaces are less negative (Fig. S5). CFU counts from samples after cytochrome C addition showed no differences in cell numbers, and therefore, it is unlikely that bacterial cell numbers influenced cytochrome C binding (Fig. 7B; Fig. S5). These data indicate that the lack of T7SS effector EsxC impacts the surface charge of *S. aureus*.

*S. aureus* has been reported to resist daptomycin through possessing a more positive surface charge (31, 32). Based on the increased negative surface charge observed for the T7SS mutants, we hypothesized that daptomycin could bind more easily in the absence of a functional T7SS. To test this, BODIPY-tagged daptomycin was used to quantify binding via fluorometry (Fig. 7C) and fluorescence microscopy (Fig. 7D). In the presence of daptomycin, a higher level of daptomycin was observed to bind around the septum for the WT as reported previously (33), while Δ*esxC* showed a significantly higher fluorescence. Thus, our data suggest that reduced electrostatic repulsion of daptomycin may contribute to the increased daptomycin binding in Δ*esxC*.

### Calcium affects the growth of Δ*esxC* and sensitivity to daptomycin

**Fig 8:**
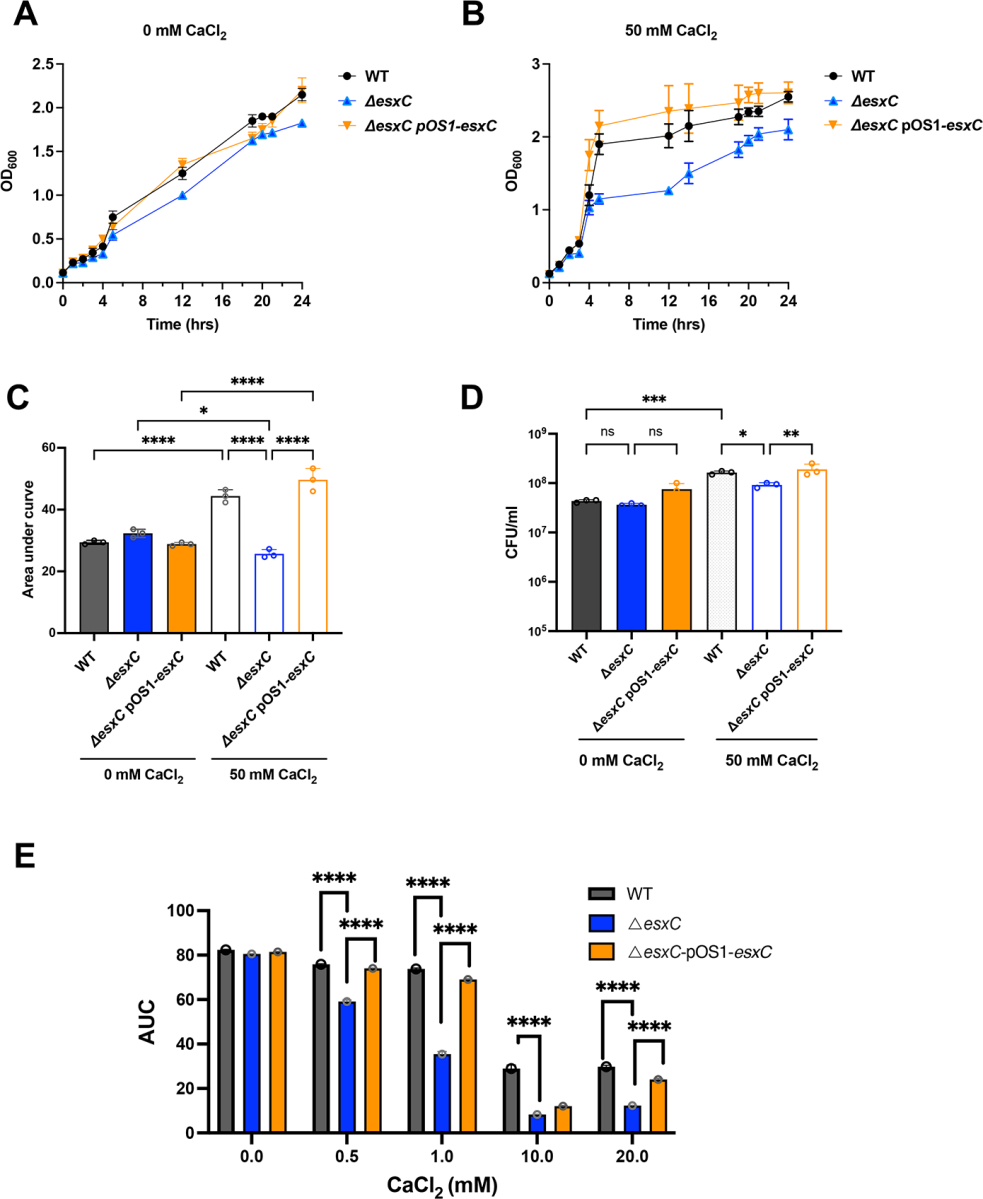
Calcium affects growth of *esxC* mutant and daptomycin sensitivity growth curves of *S. aureus* WT USA300 JE2, Δ*esxC*, and Δ*esxC* pOS1-*esxC* in synthetic minimal medium in the absence (**A**) or presence (**B**) of 50 mM CaCl_2_, mean ± SEM is shown. (**C**) AUC was calculated from (**A and B**) and the mean AUC ± SD was plotted. Mean ± SD, *P = 0.011, ***P < 0.001, using ordinary one-way ANOVA with Tukey’s multiple comparisons test, (**D**) CFU was enumerated after 5 h growth in absence or presence of 50 mM CaCl_2_., mean ± SD, *P = 0.03, **P = 0.001, ***P = 0.0004, ns - non-significant using ordinary one-way ANOVA, with a multiple Tukey’s comparisons test. (**E**) *S. aureus* WT USA300 JE2, Δ*esxC*, and Δ*esxC* pOS1-*esxC* were grown in TSB supplemented with increasing calcium chloride concentrations (0 to 20 mM) in the presence of daptomycin (5 µg/mL). AUCs calculated from growth curves shown in Fig. S6 are shown. mean ± SD, ***P = 0.0001, ****P < 0.0001, using two-way ANOVA with a Tukey’s multiple comparison test.

Calcium is an important component in membranes and affects both structure and charge of bacterial membranes (34). Calcium is also required for the activity of daptomycin and can enhance killing by gramicidin (35, 36). We hypothesized that EsxC may control membrane integrity through modulating the membrane binding of calcium. To test this, EsxC mutants were cultured in minimal medium with or without calcium chloride (50 mM). While both WT and mutant strains showed slow growth in minimal medium in the absence of calcium, in the presence of 50 mM calcium chloride, the mutant grew much slower than the WT and the *esxC*-complemented strains, with lower CFUs recovered after 4 h growth (Fig. 8A through D). We then examined if the daptomycin toxicity was dependent on the concentration of calcium available. WT, Δ*esxC*, and the complemented strains were cultured in the presence of 5 μg/mL daptomycin and increasing calcium chloride concentrations (0 to 20 mM). While daptomycin did not have any effect in the absence of calcium as expected, a dose-dependent decrease in growth of the WT strain was observed in CaCl_2_, with the WT showing slower growth at 10 and 20 mM CaCl_2_ compared with 1 mM (Fig. 8E; Fig. S6). The mutant was more sensitive to daptomycin at all calcium concentrations compared with the WT and complemented strains, with increased growth inhibition at higher CaCl_2_ concentrations.

Hence, calcium binding to membranes and/or downstream signaling may be impacted in the Δ*esxC*, affecting its ability to grow in the presence of calcium and its sensitivity to membrane-targeting drugs like daptomycin.

### Decreased intracellular survival of Δ*esxC* in the presence of daptomycin

**Fig 9:**
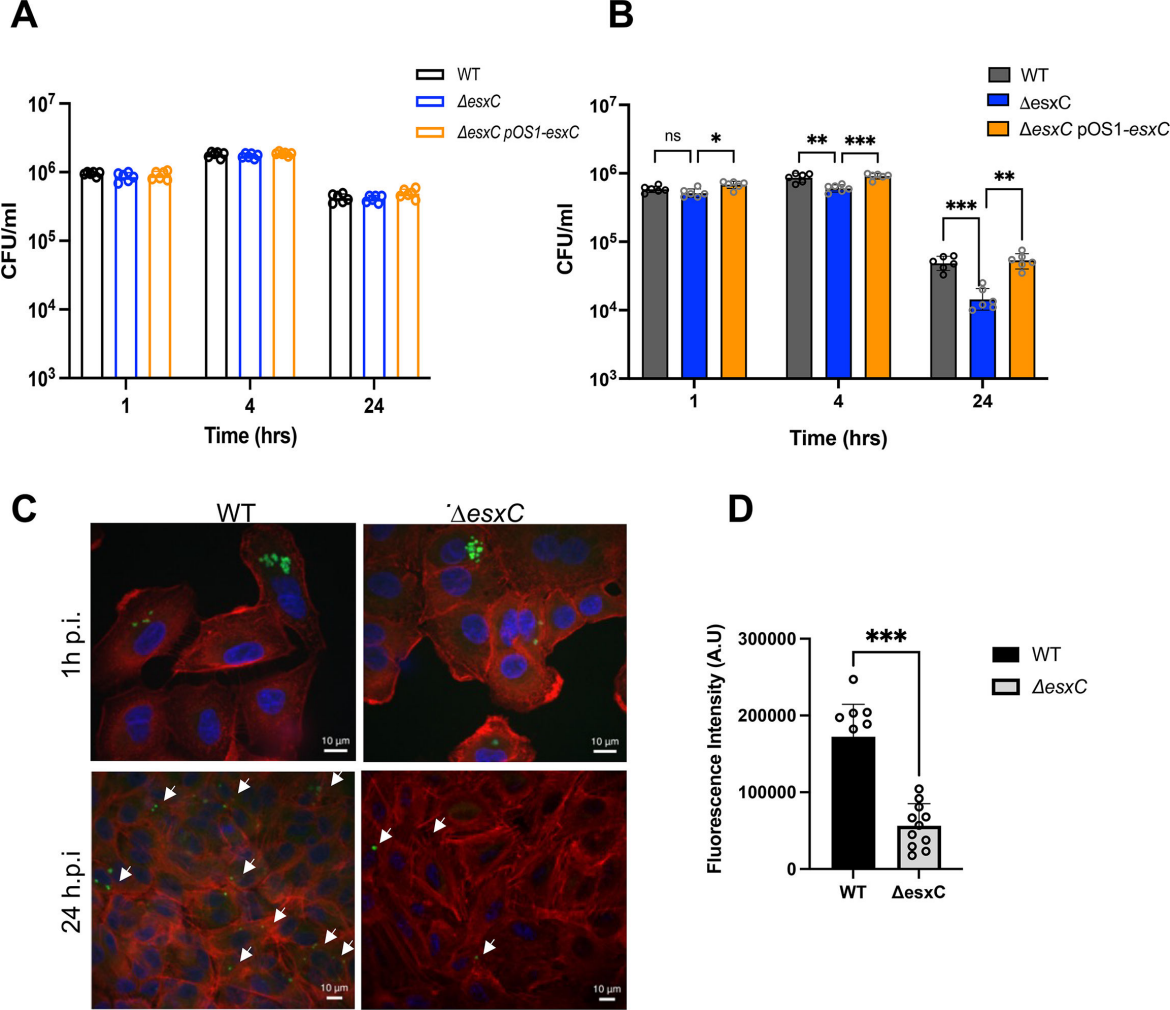
Presence of EsxC affects intracellular bacterial killing by daptomycin. CFU counts at various time points during epithelial cell infection assay with WT USA300 JE2 and Δ*esxC* after 1, 4, and 24 h infection in the absence (**A**) or in the presence of 20 μg/mL daptomycin (**B**), *N* = 3 (biological replicates), mean ± SD is shown, *P = 0.01, **P = 0.004, ***P = 0.002, one way ANOVA with Tukey’s multiple comparison tests. (**C**) Representative confocal micrographs of cells after 1 h or 24 h post-infection. DAPI (blue), F-actin (red), and *S. aureus* (green, white arrows). (**D**) Confocal image quantification from five images each from three independent experiments after 24 h. Mean ± SD of intensity is shown, ***P < 0.001 using an unpaired *t*-test.

Daptomycin has been shown to kill intracellular *S. aureus* less effectively (37). To determine if deletion of *esxC* from *S. aureus* would enhance intracellular killing activity of daptomycin, A549 human lung epithelial cells (16) treated with a high concentration of daptomycin (20 μg/mL) or untreated were infected with *S. aureus*. There were no significant differences in bacterial internalization or in intracellular bacterial survival between WT and Δ*esxC* in untreated epithelial cells (Fig. 9A). In the daptomycin-treated cells, while there were no differences between numbers of internalized WT and Δ*esxC* 1 h post infection (p.i.), a significant decrease was observed in Δ*esxC* survival within cells treated with daptomycin in comparison to WT, 4 h, and 24 h p.i. (Fig. 9B). Notably, there was a significant number of intracellular WT *S. aureus* surviving after treatment with daptomycin. Confocal microscopy of infected cells also showed more *S. aureus* WT at 24 h p.i. compared with Δ*esxC* in the presence of daptomycin (Fig. 9C and D). WT and mutant strains had similar growth rates in DMEM-10 (Fig. S7A), with a growth defect in the presence of daptomycin as expected (Fig. S7B). Therefore, our data suggest that daptomycin is more effective in killing intracellular *S. aureus* in the absence of T7SS.

### *S. aureus* lacking EsxC is sensitive to daptomycin in a skin infection model

**Fig 10:**
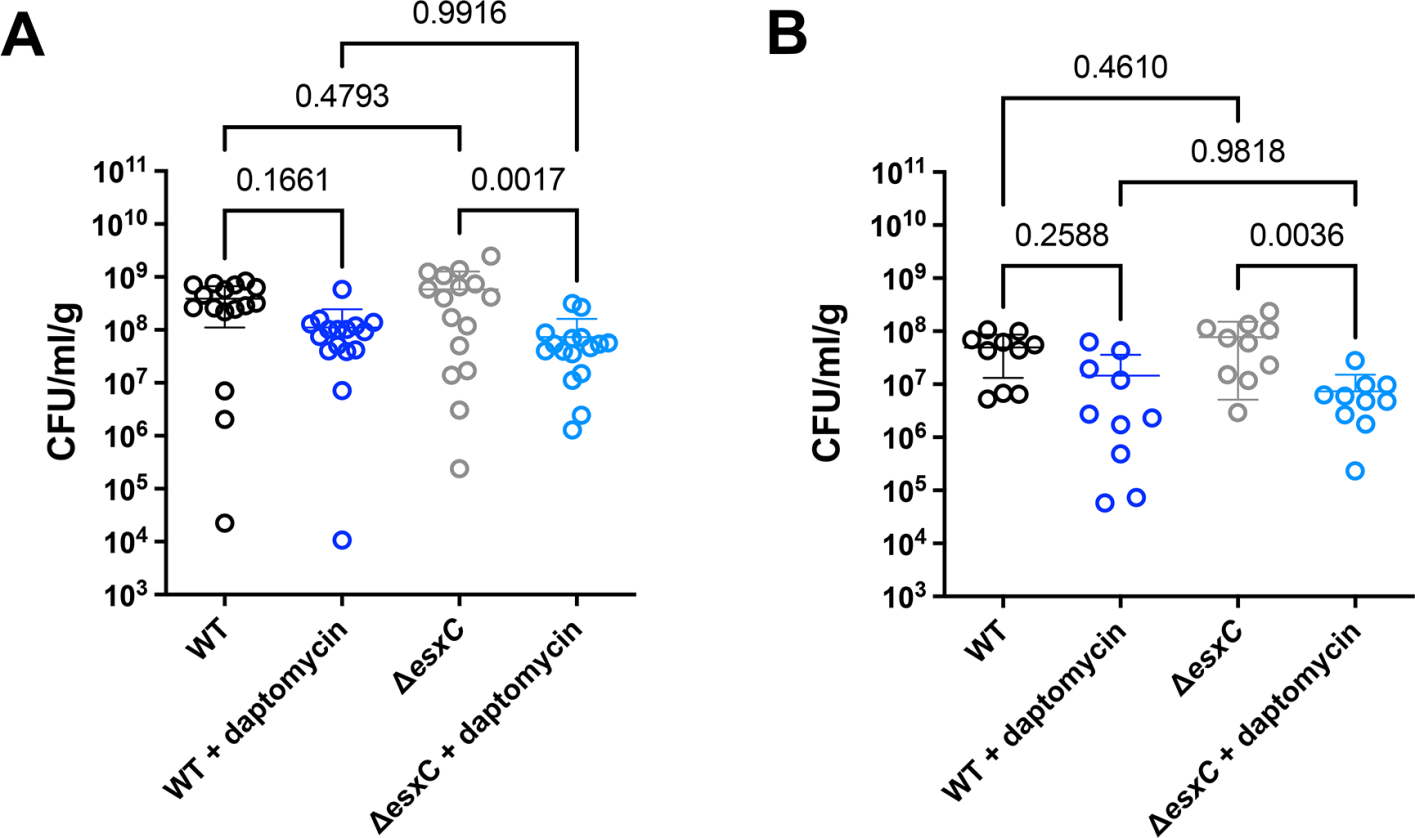
Effect of daptomycin on *esxC* mutants *in vivo.* Mice were infected in the skin with WT USA300 and Δ*esxC* mutant, followed by treatment with 10 mg/kg daptomycin. (**A**) CFU/g skin tissue was counted 24 h p.i. (*n* = 8, two lesions/animal). Graphs show mean ± SD, and a one-way ANOVA with a Tukey’s multiple comparisons test was used to determine significance. *P*-values are indicated in the graph. (**B**) CFU/g was calculated 72 h p.i. (*n* = 5, two lesions/animal), mean ± SD shown, one-way ANOVA with Tukey’s multiple comparisons test; *P*-values are indicated in the graph.

As T7SS proteins have been reported to be important in various murine infection models (6, 8, 38), we next investigated effects of daptomycin on *S. aureus* infection and the impact of EsxC on daptomycin killing *in vivo* employing a localized murine skin abscess model. BALB/c mice were infected with *S. aureus* WT and Δ*esxC* intradermally, followed by treatment with 10 mg/kg daptomycin as described in Materials and Methods. In the non-treated groups, bacterial counts from the site of infection (lesions) showed no significant difference in bacterial numbers between WT- and Δ*esxC-* infected mice after 24 or 72 h of infection. There were also no differences in bacterial survival between WT- and Δ*esxC-*infected mice treated with daptomycin. A significant decrease was, however, seen in the numbers of Δ*esxC* present in the skin of daptomycin-treated mice compared to the untreated group 24 and 72 h p. i., while this was not the case for the WT-infected group. (Fig. 10A and B). No significant differences were observed between the areas of lesions measured for WT or Δ*esxC* after 24 or 72 h infections and in daptomycin-treated mice (Fig. S8). Although the dose of daptomycin used in the timeframe of treatment was not sufficient to treat WT *S. aureus* infection, these data suggest that the strain lacking EsxC was more susceptible to daptomycin *in vivo* compared with the WT.

## DISCUSSION

The *S. aureus* T7SS has been strongly associated with staphylococcal virulence and the ability of this pathogen to persist during infection (7). Its importance for bacterial competition between *S. aureus* strains has been recently highlighted (13). However, the role of this system in staphylococcal physiology has been poorly defined. Here, we report that EsxC, a T7SS effector, is important for maintaining cell membrane homeostasis through modulating membrane properties including fluidity, accompanied by changes in membrane protein localization, rate of cell wall synthesis, and surface charge. Our data indicate that EsxC may mediate these membrane effects through modulating calcium binding to the membrane. Consequently, the lack of EsxC impacts activity of membrane-acting drugs including daptomycin, a key drug used to treat resilient staphylococcal infections, with modulation of the activity of daptomycin both intracellularly as well as during infection *in vivo*. Although we observe T7SS effector specificity in daptomycin sensitivity and membrane defects, as EsxC secretion is co-dependent on other T7SS effectors, EsxC may mediate these effects in association with other T7SS proteins.

Several studies in mycobacterial species have linked the T7SS to cell surface integrity in different ways (20, 39–42). The ESX-1 system has been previously reported to impact cell membrane permeability in *M. tuberculosis*, although this effect appeared to be strain specific (39). However, ESX-5, which is considered essential for *in vitro* growth, has been linked to mycobacterial capsular, cell wall, and outer membrane permeability (20, 40, 43) and proposed to mediate transport of cell envelope proteins required for uptake of essential nutrients to the outer membrane. In *S. aureus,* we previously reported that the T7SS components EsxC and EssC were associated with the maintenance of membrane integrity in response to antimicrobial host fatty acids (22). We and others have previously reported that some secreted T7 effectors like EsxC and EsxA are associated with the staphylococcal cell membrane and/or cell wall (8, 22, 44). T7SS has also been previously linked to membrane fluidity; higher levels of T7SS transcription were associated with a decrease in membrane fluidity, caused by the fatty acid kinase complex, which incorporates host fatty acids into the membrane, inducing a change in membrane dynamics (27).

It is important to consider that T7SS substrates are secreted in a co-dependent manner, so EsxC-mediated effects may be attributed to another T7SS substrate like EsxA and LXG toxins like EsaD (11, 13). EsxC was reported to be part of the EsaD secretion complex, along with EsxD and EsxB, with EsxD required for EsaD secretion (15). However, mutants lacking EsxB and EsxD did not show similar phenotypes to EsxC. EsxD and EsxB did not appear to be associated with surface morphology defects by SEM, surface charge, and daptomycin sensitivity, indicating that these substrates did not play a role in the membrane homeostasis. Hence, at least in USA300, EsaD may not be involved, and EsxC likely has distinct effects. However, EsaD was depleted in *esxC* mutant membranes and supernatants. We cannot rule out that EsaD contributes to the defects seen. On the other hand, common phenotypes were seen for strains, which lacked EsxC or EsxA. Previous reports have suggested that EsxC and EsxA can interact (11), and decreased EsxA was seen in the Δ*esxC* secretome, indicating co-dependent secretion of EsxA and EsxC as expected (8, 11, 22). Therefore, interactions between EsxC and EsxA may be important in modulating membrane integrity. While *esxA* is conserved across all *S. aureus* strains, *esxC* is only found in the *essC1* locus (8, 9). It is, however, likely that all *S. aureus* strains encode a protein orthologous to EsxC as proteins in module 2, with variants essC2-4, have not yet been characterized and could function like EsxC. Finally, in the RN6390 strain background, *esxC* and *essC* did not show similar differences to the USA300 strains by SEM, which may be due to differences in global regulatory circuits that may control cell wall synthesis (Agr, Sar) (45–47).

To explain the EsxC-associated membrane defects, multiple possibilities were considered. First, EsxC could affect other membrane-associated proteins, impacting lipid packing and fluidity of the membrane; EsxC could be a chaperone for membrane proteins with a role in targeting them to the membrane. A recent bacterial two-hybrid library screening we performed, however, was not successful in identifying any direct bacterial interactors, although such interactions may be transient. Second, it is possible that EsxC may impact the state of the membrane by binding other molecules like metal ions required for maintaining stability of the membrane (48). Our data indicate that the T7SS substrates may be involved in modulating metal binding to the membrane.

Calcium and other metal ions like magnesium are known to impact structure and function of bacterial membranes (34, 49, 50). Calcium maintains bacterial outer membrane structure through binding to bacterial lipopolysaccharide and also influences bacterial membrane fluidity (51, 52). Calcium can also have antibacterial effects; higher concentrations of calcium can disrupt membrane integrity (50, 53, 54). Calcium affects the growth of *S. aureus* lacking EsxC, indicating that the protein may modulate calcium binding to the membrane. EsxC may directly or with other T7SS effectors or membrane proteins, bind to calcium, and/or be involved in transporting or modulating calcium binding to the cell envelope. The molecular mechanisms underlying calcium interactions of EsxC/ other T7SS substrates need further investigation.

Daptomycin is thought to bind to the cell membrane before forming a complex with cell wall biosynthesis machinery, preventing cell wall synthesis, before dispersing throughout the bacterial membrane causing depolarization (55). Daptomycin, although not a cationic antimicrobial peptide, forms a highly positively charged complex with calcium to become active (55). Increased daptomycin binding to Δ*esxC* compared with the WT suggests that the decreased positive surface charge of Δ*esxC* allows easier daptomycin binding to the cell membrane. In agreement with this, T7SS substrates may influence the free calcium available for activating daptomycin. Daptomycin also affects bacterial membrane fluidity through perturbing membrane microdomains (56), and clinical daptomycin-resistant isolates have generally been associated with increased fluidity (57, 58). Thus, EsxC could further contribute to daptomycin resistance by altering membrane fluidity and charge. Daptomycin resistance in *S. aureus* has been mainly associated with mutations in genes encoding for multipeptide resistance factor (MprF), a lipid biosynthetic enzyme, and the two-component system, vancomycin-resistance associated sensor/regulator (VraSR) (59–61). Proteomic analysis of mutant whole lysates did not show any modulation of proteins implicated in daptomycin resistance, and genome sequencing did not reveal any changes in the Δ*esxC* genome (22).

In contrast to daptomycin, bithionol and gramicidin are uncharged (24, 25); therefore, surface charge and decreased electrostatic repulsion of antibiotics is not solely responsible for increased Δ*esxC* sensitivity, although calcium is known to increase gramicidin activity (35). As all three antimicrobials have different mechanisms of action once bound to the membrane, before causing membrane depolarization, the subsequent steps after membrane binding appear to be important for efficacy. Therefore, daptomycin treatment, which impacts both the cell membrane and the cell wall, may cause a larger effect in the *esxC* mutant.

While daptomycin kills Δ*esxC in vivo*, at the dose and with the strains of *S. aureus* used, there was no impact of the EsxC in bacterial survival during acute skin infection. T7SS proteins have been previously reported to be important for bacterial virulence using invasive kidney abscess, nasal colonization and skin infection models, although studies have used different *S. aureus* strains (6, 8, 38). The lack of decrease in the lesion size of the T7SS mutant-infected mice treated with daptomycin, unlike the trend towards decreased lesion size observed for the WT (Fig. S7), could indicate that the increased bacterial killing may cause an altered local immune response. A recent study reported that in a *S. aureus* strain WU1, the T7SS was important only for invasive infection and not localized nasal colonization (62).

Currently, levels of resistance to daptomycin are low; however, with increasing resistance in other last-line antibiotics, such as vancomycin, the use of daptomycin will increase going forward, likely leading to the occurrence of more resistant isolates (63). Therefore, targeting new proteins that can enhance existing treatments could be a promising approach. Our data indicate that inhibitors of T7SS proteins or of T7SS secretion may have good potential in combination drug therapies.

## MATERIALS AND METHODS

### Bacterial strains

*S. aureus* strains used in this study are included in Table 2. Strains were cultured aerobically in tryptic soy broth (TSB) (Sigma-Aldrich) at 37°C. Overnight cultures were subcultured into fresh TSB and grown to a density of optical density (OD_600_) of 1, unless stated otherwise. For complemented strains, TSB was supplemented with 10 μg/mL chloramphenicol.

**TABLE 2:** Strains and plasmids used in this study

Strain or plasmid	Description	Source or reference
*Staphylococcus aureus*		
USA300 JE2	Plasmid-cured USA300 LAC	BEI Resources (NARSA)
USA300 JE2 Δ*essC*	USA300 LAC JE2 defective for EssC	(22)
USA300 JE2 Δ*esxC*	USA300 LAC JE2 defective for EsxC	(22)
USA300 LAC	Community acquired MRSA	(64)
USA300 LAC Δ*esxA*	USA300 LAC defective for EsxA	(16)
USA300 LAC Δ*esxB*	USA300 LAC defective for EsxB	(16)
Newman	Methicillin-sensitive *S. aureus*	(64)
Newman Δ*esxA*	Newman defective for EsxA	(16)
Newman Δ*esxB*	Newman defective for EsxB	(16)
RN6390	NCTC8325 derivative, Δ*rbsU,* Δ*tcaR,* cured of φ11, φ12, and φ13	Tracy Palmer (8)
RN6390 Δ*essC*	RN6390 defective for EssC	Tracy Palmer (8)
RN6390 Δ*esxC*	RN6390 defective for EsxC	Tracy Palmer (8)
Plasmids		
pOS1	Empty vector for complementation	Olaf Schneewind (65)
pOS1-*esxC*	*esxC* complementation vector	(22)

### Growth curves

Overnight bacterial cultures were diluted to an OD_600_ of 0.1 in TSB or TSB supplemented with antimicrobials where specified. Bacteria were grown in a 96-well plate with shaking, and the OD_600_ was measured every 15 min with a FLUOstar OMEGA plate reader (BMG Labtech, UK). At appropriate time points, 100 μL of the samples was taken for CFU determination.

For growth curves in defined medium, overnight cultures were subcultured into minimal defined medium, prepared as described by Machado and colleagues (66), at an initial OD_600_ of 0.15, and supplemented with varying concentrations of CaCl_2_ where stated. Bacterial growth was monitored manually by measuring OD_600_ at different time points using a spectrophotometer (Biochrom) due to issues with increased bacterial clumping in this medium on microtiter plates. Then, 100 μL of the samples was also taken for CFU determination at different time points.

### Laurdan staining

Membrane fluidity was measured using the fluorescent dye Laurdan (Sigma) and was based on protocols described by Wenzel and colleagues (67). Then, 1 mL aliquots of logarithmic phase *S. aureus* were washed in PBS. The samples were incubated with a final concentration of 100 μM Laurdan for 5 min at 37°C in the dark, washed four times in pre-warmed PBS, following which 200 μL was transferred to pre-warmed black-walled 96-well plates. Membrane fluidity was determined by using an excitation wavelength of 350 nm and measuring fluorescence at 460 and 500 nm emission wavelengths on a BioTek Cytation 5 Cell Imaging Multimode Reader (Agilent). The generalized polarization (GP) value was calculated using the following calculation, GP = (I_460_-I_500_) / (I_460_ + I_500_).

### DiIC12 staining

Overnight bacterial cultures were diluted to an OD_600_ of 0.15 in TSB with 1 μg/mL 1,1′-didodecyl-3,3,3′,3′-tetramethylindocarbocyanine perchlorate (DiIC12) (Invitrogen). Cultures were grown to an OD_600_ of 1.0, centrifuged, and washed twice with fresh TSB. Samples were imaged on agarose pads using a Leica DMi8 widefield microscope (Leica Microsystems, UK).

### Cytochrome C binding assay

Overnight bacterial cultures were pelleted and washed with 20 mM MOPS buffer, pH 7. The cells were resuspended in MOPS buffer at an OD_600_ of 1 and incubated with 50 μg/mL cytochrome C at room temperature for 15 min. The cells were pelleted and the remaining cytochrome C in the supernatant was measured spectrophotometrically at OD_410_ using a FLUOstar OMEGA plate reader (BMG Labtech, UK).

### Invasion and survival assays

Infection assays were carried out in 24-well plates (Falcon), or RS-treated glass chamber slides (154526, Nunc, Lab Tek II). Overnight *S. aureus* cultures were diluted to an OD_600_ of 0.15 and grown to an OD_600_ of 1, resuspended in DMEM to 2.5 × 10^6^ CFU/mL. A549 epithelial cells were seeded at a density of 2.5 ×10^5^ 1day before being infected with *S. aureus* at a multiplicity of infection (MOI) of 10 for 1 h at 37°C with 5% CO_2_. To kill any extracellular bacteria, cells were washed with DMEM containing 20 μg/mL lysostaphin (Sigma-Aldrich) and 50 μg/mL gentamycin (Melford) and incubated for 30 min. Cells were washed twice with PBS and lysed with 1 mL cold sterile water before plating for CFU determination. To investigate the course of infection after extracellular bacterial killing, DMEM with 1 mM CaCl_2_ only, or DMEM with 1 mM CaCl_2_ and 10 μg/mL daptomycin were added and incubated until the desired timepoint.

### Murine skin abscess model

All animal procedures were carried out as per protocols in the Project Licence PCEC27E7D. All local ethical and home office approvals were obtained for animal experimentation. For the murine skin abscess model, female 7/8-week-old BALB/c mice (Charles River Laboratories, UK) flanks were shaved before intradermal injection with 50 μL of bacterial suspension on each flank, resulting in a final bacterial count of 1–2 × 10^6^ per mouse. Overnight *S. aureus* cultures were subcultured, grown to OD_600_ of 1, centrifuged and washed twice with PBS, and diluted in PBS to achieve a final CFU/mL of 1–2 × 10^7^ for infection. Two hours after infection, mice were injected intravenously via the tail vein with the dose of 10 mg/kg daptomycin or PBS. This was repeated once every 24 h. During the experiment, mice were weighed daily, and any abscess formation was measured and recorded. After 24 or 72 h, mice were euthanized by CO_2_ inhalation, and skin at the infection site was incised. The skin samples were weighed and homogenized in PBS using a FastPrep (MP Biomedicals) at 4 m/s for 60 s for a total of 6 cycles. Homogenates were diluted in PBS, and CFU/mL was determined. CFU counts were normalized to lesion weight.

## SUPPLEMENTAL MATERIAL

Supplemental figures jb.00380-25-s0001.pdf Figures S1 to S8.

Supplemental material jb.00380-25-s0002.pdf Supplemental methods.

Table S1 jb.00380-25-s0003.pdf Secreted proteins significantly changed in ΔesxC relative to WT USA300 JE2.

ASM does not own the copyrights to Supplemental Material that may be linked to, or accessed through, an article. The authors have granted ASM a non-exclusive, world-wide license to publish the Supplemental Material files. Please contact the corresponding author directly for reuse.
