# Supplementary material for: The staphylococcal type VII secretion system protein EsxC impacts daptomycin sensitivity through controlling bacterial cell envelope integrity
Source: J Bacteriol. 2026 Jan 12;208(2):e00380-25. doi: 10.1128/jb.00380-25 (PMC12918736; doi:10.1128/jb.00380-25)
Supplement: Supplemental figures — Figures S1 to S8. [file jb.00380-25-s0001.pdf]

Fig S1

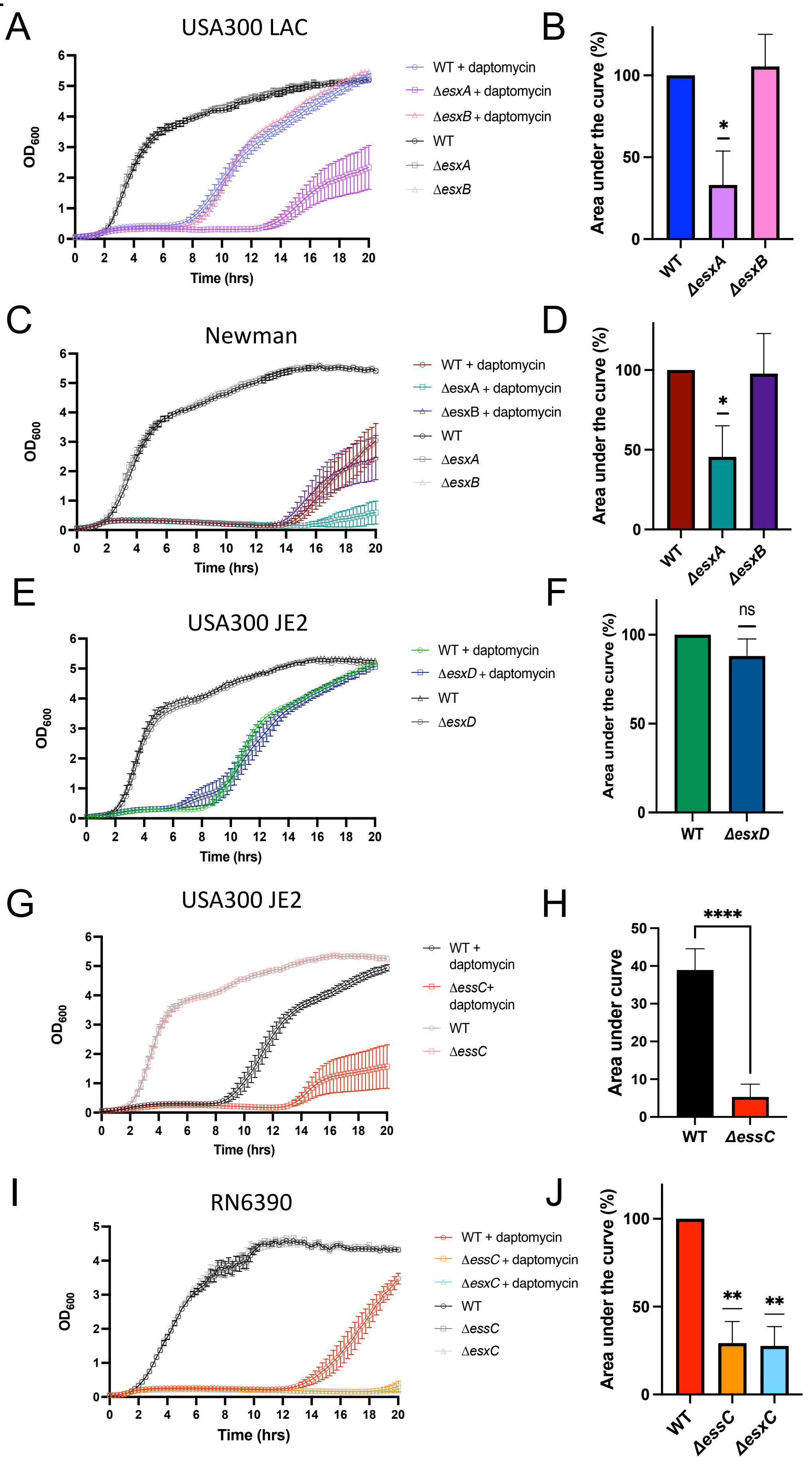
A
USA300 LAC
OD600
Time (hrs)
WT + daptomycin
ΔesxA + daptomycin
ΔesxB + daptomycin
WT
ΔesxA
ΔesxB
B
Area under the curve (%)
*
WT
ΔesxA
ΔesxB
C
Newman
OD600
Time (hrs)
WT + daptomycin
ΔesxA + daptomycin
ΔesxB + daptomycin
WT
ΔesxA
ΔesxB
D
Area under the curve (%)
*
WT
ΔesxA
ΔesxB
E
USA300 JE2
OD600
Time (hrs)
WT + daptomycin
ΔesxD + daptomycin
WT
ΔesxD
F
Area under the curve (%)
ns
WT
ΔesxD
G
USA300 JE2
OD600
Time (hrs)
WT + daptomycin
ΔessC+ daptomycin
WT
ΔessC
H
Area under curve
****
WT
ΔessC
I
RN6390
OD600
Time (hrs)
WT + daptomycin
ΔessC + daptomycin
ΔesxC + daptomycin
WT
ΔessC
ΔesxC
J
Area under the curve (%)
**
**
WT
ΔessC
ΔesxC

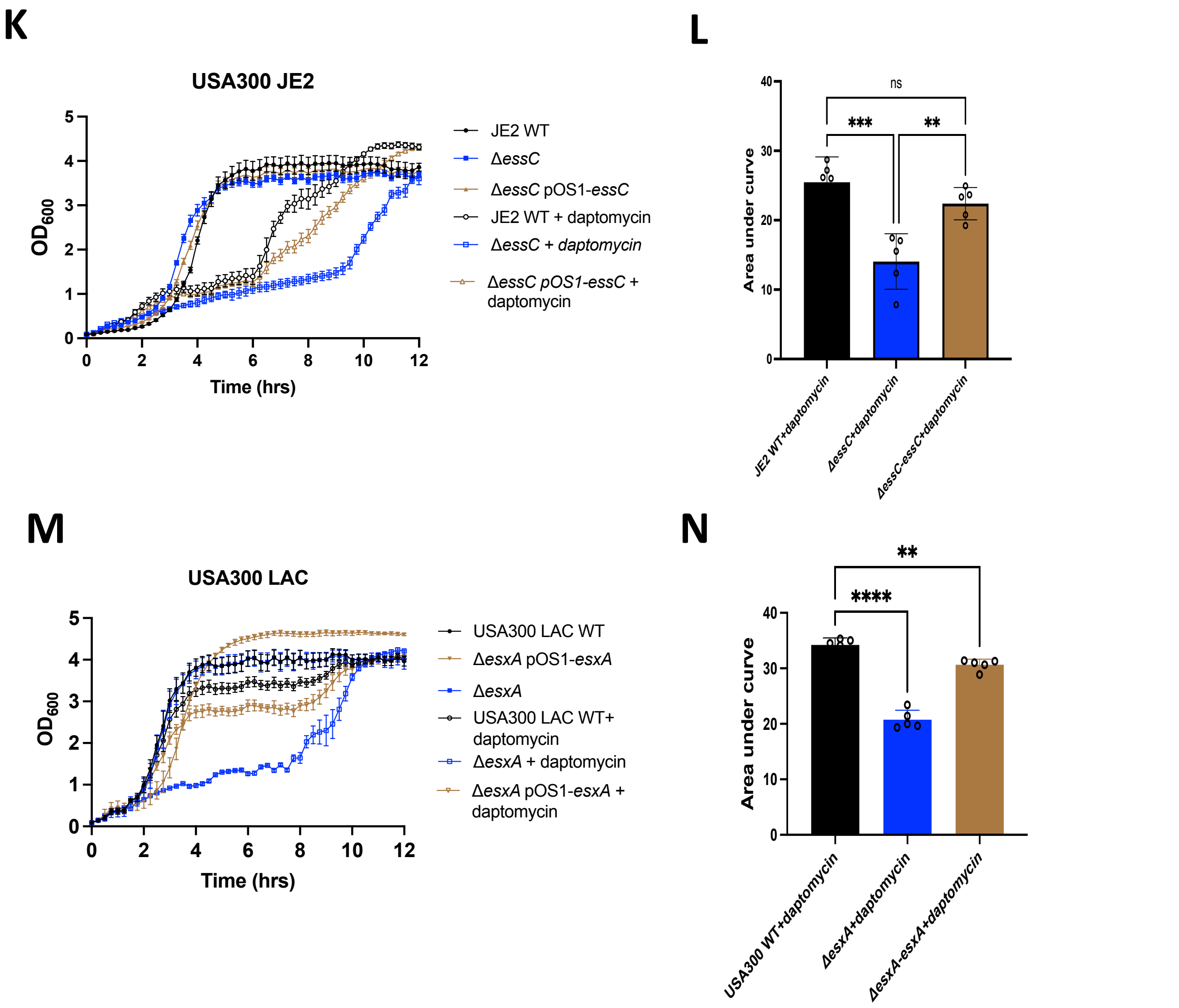

Figure S1. Growth curves in TSB for **A)** *S. aureus* USA300 LAC Δ*esxA* and Δ*esxB*, **C**) *S. aureus* Newman Δ*esxA* and Δ*esxB*, **E**) *S. aureus* USA300 JE2 WT and Δ*esxD,* **G**) *S. aureus* USA300 JE2 WT and Δ*essC,* **I**) *S. aureus* RN6390 WT, Δ*essC* and Δ*esxC*, in the absence or presence of 5 µg/ml daptomycin and 1 mM $CaCl_2$. Mean ± SEM are shown, N = 3. The AUC was calculated for the growth curves of all strains cultured in presence of daptomycin in **A, C, E, G** and **I** are presented as % relative to WT, mean ± SD is shown (**B, D, F, H, J**). * P ≤ 0.05, ** P ≤ 0.01 using a one-sample t test. **J**) shows AUC of **I**) ****P ≤ 0.0001, unpaired t test. **K**) Growth curves for USA300 JE2 WT, Δ*essC, and complemented strain* Δ*essC* pOS1-*essC* in the absence and presence of 5 µg/ml daptomycin, mean ± SD are shown N=3. **L**) The AUC was calculated for the growth curves shown in (**K**), **P=0.002, ***P= 0.0001, using ordinary one-way ANOVA, with Sidak's multiple comparisons test M). Growth curves for USA300 WT, Δ*esxA,* and complemented strain Δ*esxA* pOS1-*esxA* in presence and absence of 5 µg/ml daptomycin Mean ± SD are shown N = 3. **N**) The AUC was calculated for the growth curves shown in (**M**), **P=0.002, ****P= <0.0001, using ordinary one-way ANOVA with Dunnett's multiple comparisons test.

Fig S2

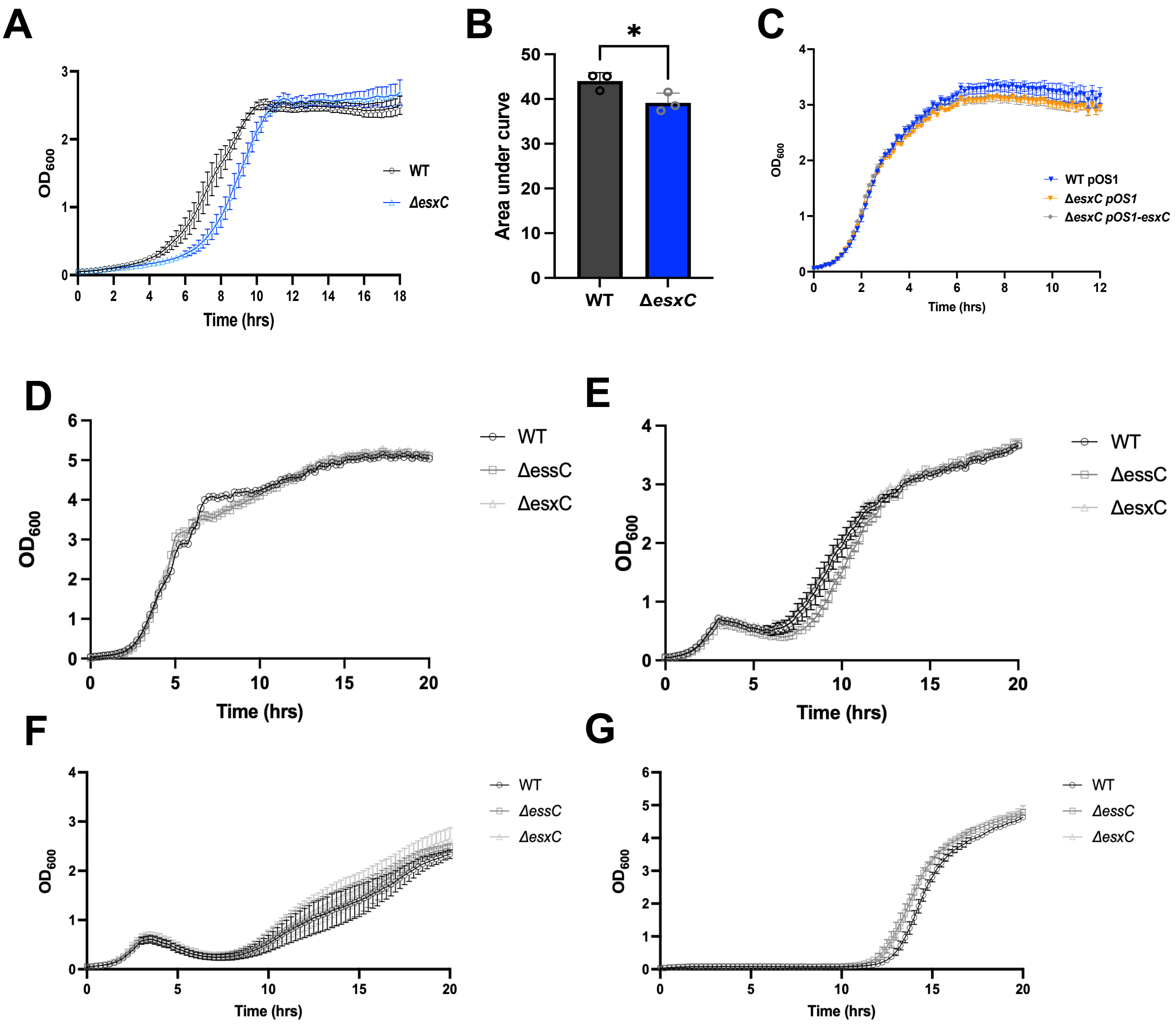

Figure S2. A) Growth curves of USA300 JE2 WT and Δ*esxC* in the presence of 1 µg/ml bithionol. Mean ± SEM, N = 3 (biological replicates). **B**) AUC were calculated and the mean AUC + SD was plotted for growth curves in (**A**). *P ≤ 0.05 using an unpaired t-test. **C**) WT, Δ*esxC and* Δ*esxC* pOS1 complemented strains in the presence of 0.5 µg/ml bithionol, Mean ± SEM, N = 3 (biological replicates). Growth curves of USA300 JE2 WT, *ΔessC* and Δ*esxC* in the presence of (**D**) 4 µg/ml ciprofloxacin (**E**) 0.5 µg/ml mitomycin C. (**F**) 1 µg/ml oxacillin. (**G**) 8 µg/ml vancomycin. For all growth curves mean and SEM are shown, N = 3 (biological replicates).

Fig S3

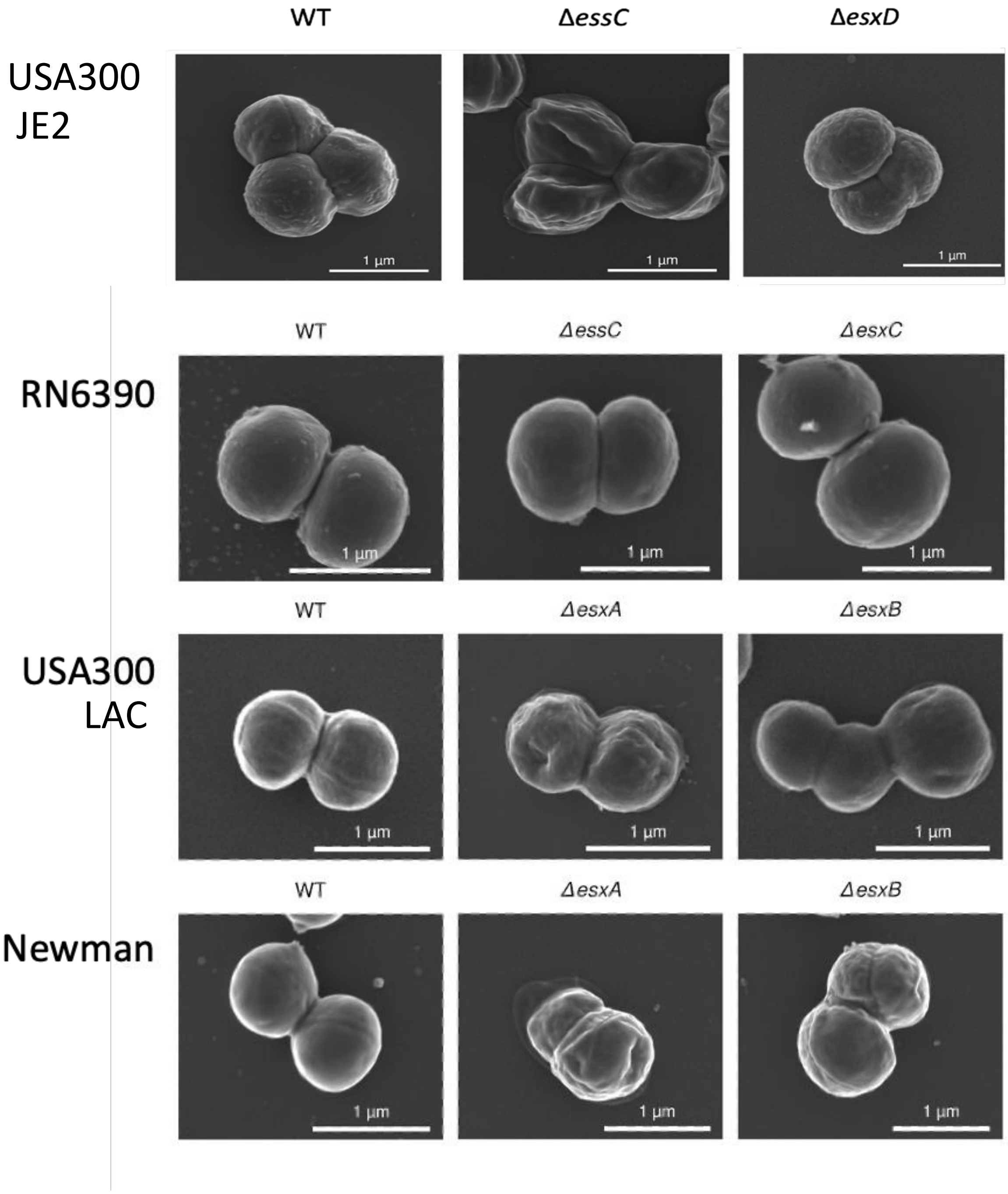

Figure S3. Scanning electron micrographs of *S. aureus* RN6390, USA300, Newman and their T7SS mutants**.**

Fig S4

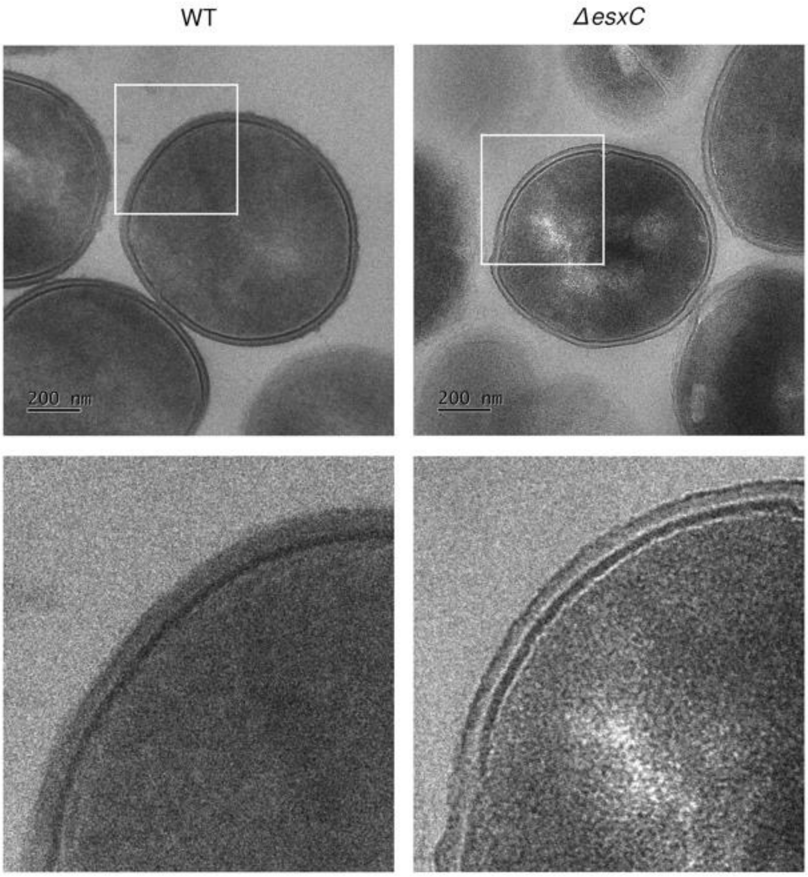

Figure S4. Transmission electron micrographs of *S. aureus* USA300 JE2 WT and Δ*esxC* grown to early logarithmic phase

Fig S5

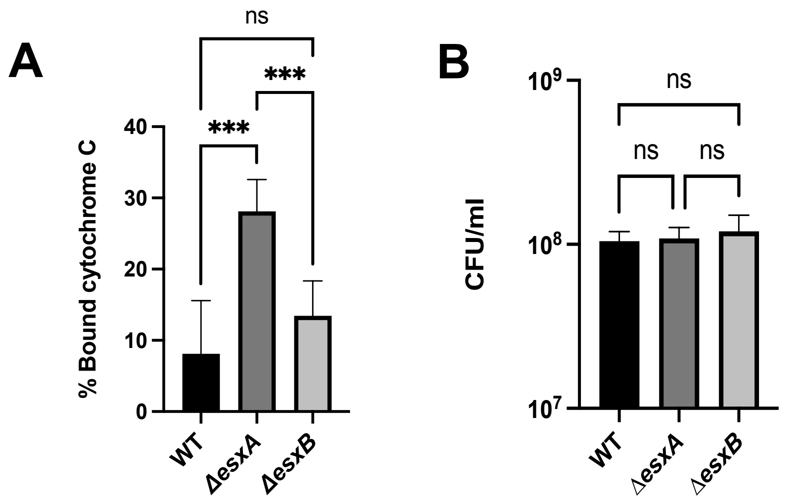

Figure S5 Quantitative binding assay of cytochrome C to USA300 LAC WT, Δ*esxA* and Δ*esxB* (**A**). The CFU of the starting inoculum was calculated for each mutant **(B)**. Mean ± SD shown, N = 3 (biological replicates), ***P < 0.001 using a one-way ANOVA with Tukey's multiple comparisons test

Figure S6

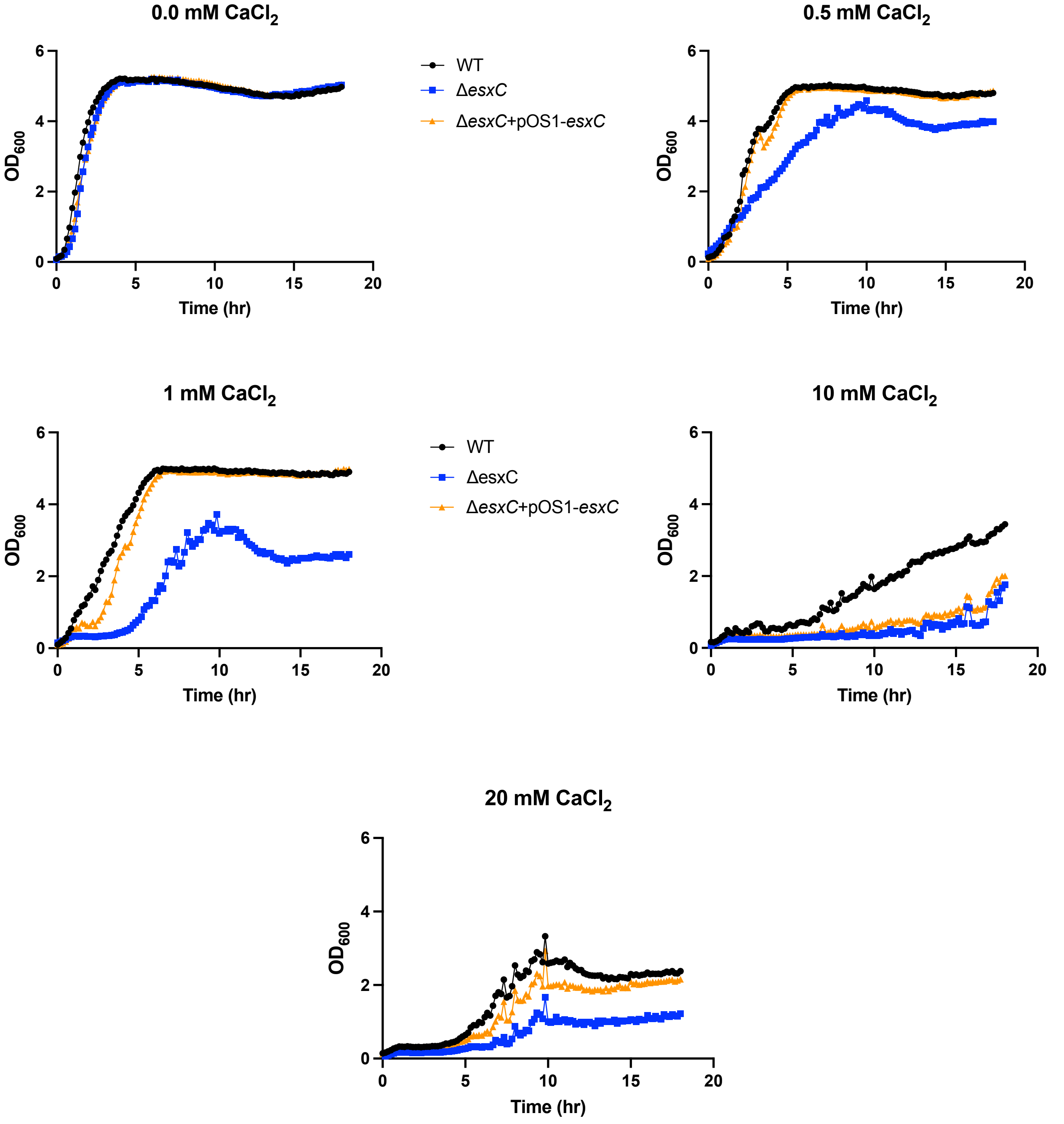

Figure S6. Growth curves of WT, *ΔesxC*, *ΔesxC* pOS1-esxC in TSB supplemented with increasing calcium chloride concentrations (0 to 20 mM) in the presence of 5 µg/ml daptomycin. Mean +SE is shown

Fig S7

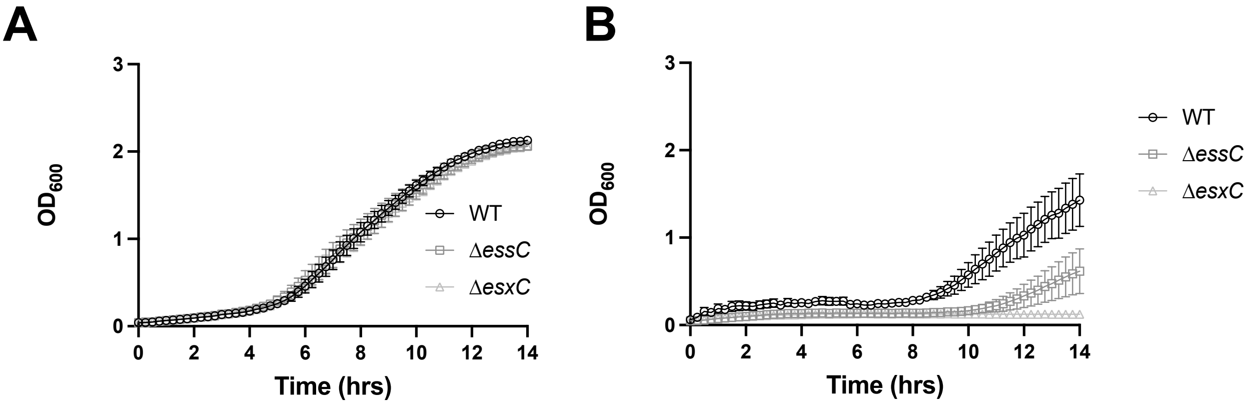

Figure S7. (A) Growth curves of WT, Δ*essC* and Δ*esxC* in DMEM + FBS. **(B)** Growth curves of WT, Δ*essC* and Δ*esxC* in DMEM + FBS in the presence of 1µg/ml daptomycin and 1 mM $CaCl_2$. Graphs show mean and SEM of three independent experiments.

Fig S8

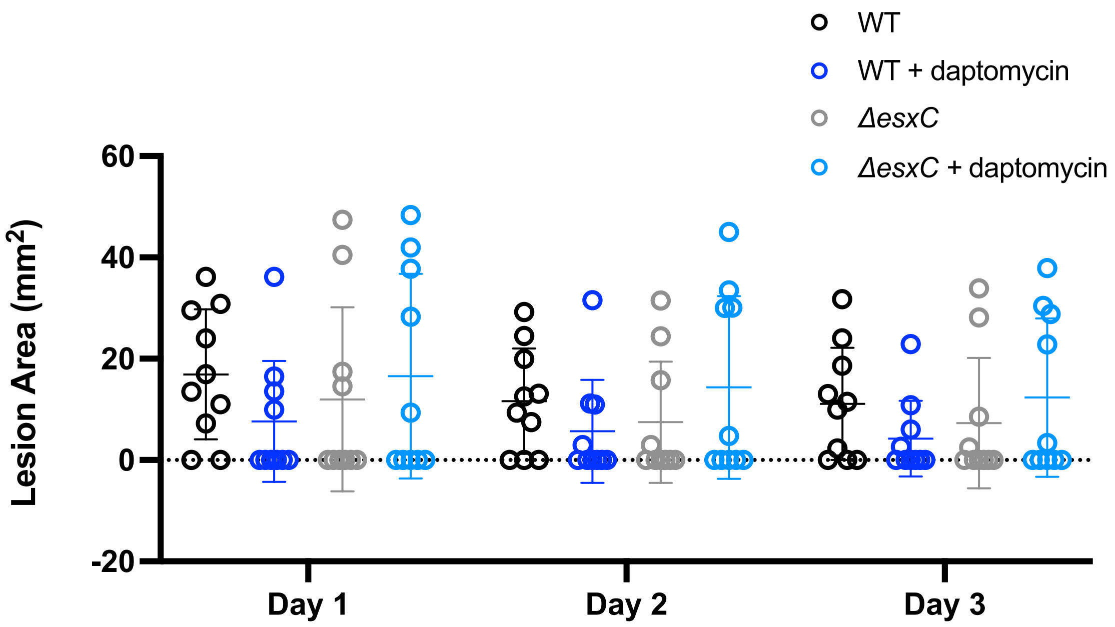

Figure S8 The area of lesions was measured and calculated daily from mice infected with *S. aureus* WT or esxC mutants. Area of lesions was measured and calculated daily. Graph shows mean ± SD (n = 5, 2 lesions/animal). Two-way ANOVA between groups revealed no significant differences between treatments.
