# Supplementary material for: The staphylococcal type VII secretion system protein EsxC impacts daptomycin sensitivity through controlling bacterial cell envelope integrity
Source: J Bacteriol. 2026 Jan 12;208(2):e00380-25. doi: 10.1128/jb.00380-25 (PMC12918736; doi:10.1128/jb.00380-25)
Supplement: Supplemental material — Supplemental methods. [file jb.00380-25-s0002.pdf]

Supplementary methods:

Daptomycin killing assays: Bacterial cultures were grown overnight in TSB, diluted to an OD_{600} of 0.15 and grown to log phase. Bacterial cultures were then treated with 10 µg/ml of daptomycin (Acros Organics) in the presence of 1 mM $CaCl_2$ (Fisher Scientific) and incubated at 37ºC for a further 2 h. Aliquots were removed at indicated timepoints, serially diluted in PBS and plated on tryptic soy agar (TSA) for CFU determination.

Pyrene decanoic acid staining: Overnight bacterial cultures were diluted to an OD_{600} of 0.15 in TSB and were grown to an OD_{600} of 1.0. Bacteria were washed with PBS prior to treatment for 30 min at 37ºC with 37.5 µg/mL lysostaphin in PBS containing 20% sucrose. The spheroblasts were then centrifuged at 8000 × g for 10 min, and the pellet resuspended in the labelling solution (PBS, 20% sucrose, 0.01% F-127, 5 µM pyrene decanoic acid). The incubation in the dark was done for 1 h at 25ºC under gentle rotation. PBS supplemented with 20% sucrose was used to wash the stained spheroblasts that were afterwards transferred to 96-well plates for fluorescence 450 nm measurements as previously described (27).

Scanning Electron Microscopy: Overnight bacterial cultures were diluted to an OD_{600} of 0.15 and grown to an OD_{600} of 1 in TSB. The pellet was washed with PBS twice before dropping onto poly-lysine coated 12 mm circular coverslips. Samples were fixed with 2.5% glutaraldehyde in PBS for 1 h at 4°C. The bacterial cells were dehydrated by washing with a series of ethanol solutions (20%, 50%, 70%, 90% and 100%) and dried with hexamethyldisilazane. The samples were sputter coated with carbon and then observed using a Gemini SEM 500 (Zeiss).

Transmission Electron Microscopy: Overnight bacterial cultures were diluted to an OD_{600} of 0.15 and grown to an OD_{600} of 1 in TSB. The pellet was washed in PBS three times and fixed with paraformaldehyde and glutaraldehyde for 1 h at room temperature (RT). Pellets were rinsed a further three times in PBS before incubation with 1% osmium tetroxide for 1 h

at RT. The bacterial cells were dehydrated by washing with a graded acetone series for 20 min each and transferred to graded acetone epoxy resin mixture for 45 min each. Cells were incubated in pure resin for 24 h before being hardened overnight at 60°C. The specimens were sectioned into 90 nm slices using an ultramicrotome (RMC) before being placed onto grids and stained with heavy metals. The samples were visualised using a TEM-2100Plus (Jeol).

Synthesis of Daptomycin BODIPY: A 1 mg/ml stock solution of 1-ethyl-3-(3-dimethylaminopropyl) carbodiimide hydrochloride (EDC), N-Hydroxysuccinimide (NHS), daptomycin and BODIPY was prepared in DMSO. Th mixture was stirred for 16-24 h at RT. 10 μl of the crude sample was removed and diluted with High Performance Liquid Chromatography (HPLC) solvent, filtered and used for HPLC analysis. The solution was purified by dialysis with ultrapure water to remove EDC, NHS and any other unwanted by-products with a membrane cut-off of 100 – 500 Da. The water was replaced every 2 h for a total of 2 times. The sample was freeze dried to remove the water.

Daptomycin BODIPY binding assay*:* *S. aureus* was grown as previously described and treated with 1μg/ml daptomycin-BODIPY once logarithmic phase had been reached. The samples were incubated at 37ºC for 30 min in the dark, then washed with PBS three times. 200 μl were aliquoted into black 96 well plates in triplicate and fluorescence was measured using a BioTek Cytation 5 Cell Imaging Multimode Reader (Agilent). The excitation wavelength used was 488 nm and emission wavelength used was 530 nm.

HADA incorporation assay

Peptidoglycan synthesis was investigated by measuring HCC-amino-D-alanine (HADA) incorporation. Overnight *S. aureus* cultures were diluted into TSB supplemented with 25 μM HADA and incubated at 37ºC in the dark. When OD_{600} of 1 was reached cultures were washed three times and fixed with 4% PFA. Alternatively, cultures were treated with daptomycin for 30 min. before washing and fixing. Microscopy was undertaken using the DAPI filter set on a Leica DMi8 widefield microscope.

Proteomics: preparation of membrane fractions: *S. aureus* strains were grown to an OD_{600} of 3.0 in TSB. After centrifugation of cultures, pellets were washed with PBS and resuspended PBS supplemented with one cOmplete™, Mini, EDTA-free Protease Inhibitor Cocktail tablet, 250 mM sucrose, 1 mM EDTA, and 50 µg/mL lysostaphin. Samples were incubated for 15 min at 37ºC. Samples were aliquoted into lysing matrix B tubes (MP Biomedicals™), and bacteria were lysed with a Fastprep machine (6.5 m/s, 2 x 45 s). Samples were centrifuged at 11 000 × *g* for 15 min, and supernatants were collected and then ultracentrifuged (230 000 × *g* for 30 min at 4ºC) in clean, thick wall polyallomer tubes. Pellets were resuspended in 400 µL PBS supplemented with 250 mM sucrose and 10% glycerol.

Preparation of culture supernatant: *S. aureus* strains were grown to an OD600 of 3 in TSB. After centrifugation of cultures, supernatants were sterile-filtered and incubated at 4°C overnight with 10% trichloroacetic acid and 50 µM sodium deoxycholate in presence of one cOmplete™, Mini, EDTA-free Protease Inhibitor Cocktail tablet (Sigma-Aldrich, UK). The precipitated proteins were centrifuged at 10 000 g at 4°C for 15 min, gently washed with acetone twice, dried at RT for 10 min and resuspended in PBS. After denaturation, precipitated proteins were run on a gel until all the proteins had moved from the stacking gel into the resolving gel. The gel was then stained with InstantBlueTM (Sigma-Aldrich, UK) for 3 h, after which the protein bands were excised and diced. Proteins were in-gel digested with trypsin as described elsewhere(64). Briefly, proteins were reduced and alkylated for 5 min at 70°C with 10 mM TCEP [tris(2-carboxyethyl) phosphine] and 40 mM CAA (2-chloroacetamide), respectively. Tryptic digestion was carried out at O/N at 37°C in 50 mM ABC.

Label-free protein quantification: Peptides prepared for proteome analyses were desalted and concentrated with a C18 cartridge in 40 µL MS buffer (2% acetonitrile plus 0.1% trifluoroacetic acid). For each sample, 20 µL were analysed by nanoLC-ESI-MS/MS using the Ultimate 3000/Orbitrap Fusion instrumentation (Thermo Scientific), and a 90-minute LC separation on a 50 cm column. The data were used to interrogate the Uniprot *Staphylococcus aureus* USA300 database UP000001939, and the common contaminant

database from MaxQuant (Cox et al., 2014). MaxQuant software was used for protein identification and quantification using 567 default settings. Intensities were log2-transformed with the Perseus software, and proteins with one or no valid value for every sample in triplicate were filtered.

The proteomics data have been deposited to the ProteomeXchange Consortium via the PRIDE partner repository with the dataset identifier PXD047096 and 10.6019/PXD047096. Membrane proteomic and secretome samples are labelled MS18-178 and MS19-126, respectively.

Confocal microscopy: At required timepoints A549 cells infected with *S. aureus* were washed twice with PBS and fixed with 4% paraformaldehyde (PFA) for 15 min. The chamber slide wells were washed in 1 ml PBS and permeabilised with 1% saponin, 0.1% triton X-100 in PBS for 20 min at RT. Cells were blocked with 3% BSA in PBS for 30 min at RT, washed with 1% BSA in PBS and twice with PBS only. Cells were stained with phalloidin 650 in 1% BSA for 40 min at RT and vancomycin-BODIPY in 1% BSA for 20 min. DAPI anti-fade mounting fluid was added to the slide. Samples were imaged using a spinning disk confocal UltraVIEW (PerkinElmer).

Image Analysis: All microscopy images were analysed using FIJI (ImageJ, Version 2.0.0-rc-69/1.52p). To calculate fluorescence, a threshold value was set, and area or number of bacterial cells was used to normalise fluorescent values.

Statistical Analysis: All experiments were performed at least three independent times. Statistical analysis was performed using GraphPad Prism 9.0 (MacOS version 9.5.1). Pairwise statistical analysis was carried out by the Mann-Whitney U test or t test for two groups, or One way ANOVAs for multiple groups, followed by post-hoc Tukey's HSD tests where necessary. Significance was denoted by asterisks; * = $P < 0.05$, ** = $P < 0.01$, *** = $P < 0.001$, **** = $P <$

0.0001 and ns = not significant. The fold changes and *P* values of the proteomics data were calculated with the R package limma (64).
