# Supplementary material for: The staphylococcal type VII secretion system protein EsxC impacts daptomycin sensitivity through controlling bacterial cell envelope integrity
Source: J Bacteriol. 2026 Jan 12;208(2):e00380-25. doi: 10.1128/jb.00380-25 (PMC12918736; doi:10.1128/jb.00380-25)
Supplement: Table S1 — Secreted proteins significantly changed in ΔesxC relative to WT USA300 JE2. [file jb.00380-25-s0003.pdf]

Table S1. Secreted proteins significantly changed in Δ*esxC* relative to WT USA300 JE2.

Uniprot ID	log_2 fold change	Adjusted *P* value	Description
A0A0H2XIV9	-4.27	7.11E-10	T7SS protein EssD
A0A0H2XHT7	-2.78	7.96E-09	Phosphocarrier protein HPr
A0A0H2XH98	-1.75	1.34E-07	Hydrolase, MutT/nudix family
A0A0H2XIK2	-1.71	1.34E-07	T7SS protein EsxC
A0A0H2XI99	-1.45	0.003045	T7SS protein EsxA
A0A0H2XFA5	-1.28	4.03E-05	Putative GTP-binding protein
A0A0H2XEF1	1.55	3.40E-06	2-oxoisovalerate dehydrogenase
Q2FEF1	1.63	0.000836	Isopentenyl-diphosphate delta-isomerase
A0A0H2XKB6	1.90	0.00012	Uncharacterized protein
A0A0H2XH53	2.58	0.042723	Uncharacterized protein
